# High-Throughput Recovery and Characterization of Metagenome-Derived Glycoside Hydrolase-Containing Clones as a Resource for Biocatalyst Development

**DOI:** 10.1128/mSystems.00082-19

**Published:** 2019-06-04

**Authors:** Zachary Armstrong, Feng Liu, Sam Kheirandish, Hong-Ming Chen, Keith Mewis, Tianmeng Duo, Connor Morgan-Lang, Steven J. Hallam, Stephen G. Withers

**Affiliations:** a Genome Science and Technology Program, University of British Columbia, Vancouver, British Columbia, Canada; b Department of Chemistry, University of British Columbia, Vancouver, British Columbia, Canada; c Department of Microbiology and Immunology, University of British Columbia, Vancouver, British Columbia, Canada; d Graduate Program in Bioinformatics, University of British Columbia, Vancouver, British Columbia, Canada; e Department of Biochemistry and Molecular Biology, University of British Columbia, Vancouver, British Columbia, Canada; f ECOSCOPE Training Program, University of British Columbia, Vancouver, British Columbia, Canada; g Peter Wall Institute for Advanced Studies, University of British Columbia, Vancouver, British Columbia, Canada; United States Naval Research Laboratory

**Keywords:** CAZymes, environmental genomics, glycosidases, high-throughput characterization, high-throughput screening, metagenomics

## Abstract

Functional metagenomics is a powerful tool for both the discovery and development of biocatalysts. This study presents the high-throughput functional screening of 22 large-insert fosmid libraries containing over 300,000 clones sourced from natural and engineered ecosystems, characterization of active clones, and a demonstration of the utility of recovered genes or gene cassettes in the development of novel biocatalysts. Screening was performed in a 384-well-plate format with the fluorogenic substrate 4-methylumbelliferyl cellobioside, which releases a fluorescent molecule when cleaved by β-glucosidases or cellulases. The resulting set of 164 active clones was subsequently interrogated for substrate preference, reaction mechanism, thermal stability, and optimal pH. The environmental DNA harbored within each active clone was sequenced, and functional annotation revealed a cornucopia of carbohydrate-degrading enzymes. Evaluation of genomic-context information revealed both synteny and polymer-targeting loci within a number of sequenced clones. The utility of these fosmids was then demonstrated by identifying clones encoding activity on an unnatural glycoside (4-methylumbelliferyl 6-azido-6-deoxy-β-d-galactoside) and transforming one of the identified enzymes into a glycosynthase capable of forming taggable disaccharides.

**IMPORTANCE** The generation of new biocatalysts for plant biomass degradation and glycan synthesis has typically relied on the characterization and investigation of one or a few enzymes at a time. By coupling functional metagenomic screening and high-throughput functional characterization, we can progress beyond the current scale of catalyst discovery and provide rapid annotation of catalyst function. By functionally screening environmental DNA from many diverse sources, we have generated a suite of active glycoside hydrolase-containing clones and demonstrated their reaction parameters. We then demonstrated the utility of this collection through the generation of a new catalyst for the formation of azido-modified glycans. Further interrogation of this collection of clones will expand our biocatalytic toolbox, with potential application to biomass deconstruction and synthesis of glycans.

## Abstract (precis)

The generation of new biocatalysts for plant biomass degradation and glycan synthesis has typically relied on the characterization and investigation of one or a few enzymes at a time. By coupling functional metagenomic screening and high-throughput functional characterization, we can progress beyond the current scale of catalyst discovery and provide rapid annotation of catalyst function. By functionally screening environmental DNA from many diverse sources, we have generated a suite of active glycoside hydrolase-containing clones and demonstrated their reaction parameters. We then demonstrated the utility of this collection through the generation of a new catalyst for the formation of azido-modified glycans. Further interrogation of this collection of clones will expand our biocatalytic toolbox, with potential application to biomass deconstruction and synthesis of glycans.

## INTRODUCTION

Plant biomass offers a sustainable source of organic matter for energy and materials and an alternative to fossil fuels. However, the industrial-scale production or biorefining of fermentable sugars from plant biomass is currently limited by the lack of cost-effective and efficient biocatalysts (1). Microbes, the earth’s master chemists, which employ biocatalytic solutions to harvest energy and transform this energy into useful molecules, offer a potential solution to this problem. Microbial degradation of carbohydrates involves the use of glycoside hydrolases (GHs), which offer some of the greatest catalytic rate enhancements among enzymes (2). GHs catalyze the degradation of diverse polysaccharides, including cellulose, the most abundant terrestrial biopolymer (3); pectins; and hemicelluloses. Beyond biomass degradation, GHs play roles in cell wall remodeling (4, 5) and biofilm processing (6–8) and have also been reengineered to act as synthetic tools termed glycosynthases (9, 10). Clearly, the identification of new GH genes has the potential to improve upon both the efficacy of current biocatalysts and the generation of new biocatalysts with potential industrial or research applications.

Most efforts to increase the diversity of functionally characterized GH genes have focused on studying one or a few enzymes at a time, including several studies that have focused on the discovery of GH genes from environmental DNA (11–15). More recently, efforts utilizing large-scale gene synthesis have enabled the exploration of phylogenetic branches within a family that has not been well characterized (16). This is a promising approach applicable to many enzyme families. However, until the cost of gene synthesis drops, the scalability and price point of synthetic screening remain out of reach for a majority of research groups. Functional metagenomics screening, coupled with high-throughput enzyme characterization, can enable activity-based annotation without the uncertainty introduced when annotation is done by sequence comparison alone. Functional screens have the ability to provide a direct link between environmental genomes and their functional activities. They can also provide the ability to discover enzymes with activities that exist outside the current paradigms of gene annotation, which in turn can better inform *in silico* and synthetic biology approaches.

The aim of this study was to interrogate large-insert fosmid libraries containing environmental DNA encoding cellobiohydrolase and β-glucosidase activities, as these functions are key to the degradation of plant polysaccharides (17). To this end, libraries were interrogated with a fluorogenic activity probe (4-methylumbelliferyl β-cellobioside [MU-C]). Each fosmid contains an environmental genomic DNA insert averaging 40 kb; thus, multiple contiguous genes, although clearly not all, will be expressed in the Escherichia coli EPI300 host used in library production and screening. The resulting set of active clones revealed a diverse set of GH genes, gene cassettes, and activities and provided a tractable genomic resource enabling us to rapidly investigate the substrate specificity, mechanism, acid tolerance, and thermal tolerance of enzymes expressed by these clones. The utility of this library was then demonstrated by identifying clones encoding activity on an unnatural glycoside (methylumbelliferyl 6-azido-6-deoxy-galactoside) bearing an azido functionality. This was done with the hope of generating a glycosynthase, from an identified hit, that is capable of transferring the taggable 6-azido-6-deoxy-galactose moiety onto acceptor molecules.

## RESULTS AND DISCUSSION

**FIG 1:**
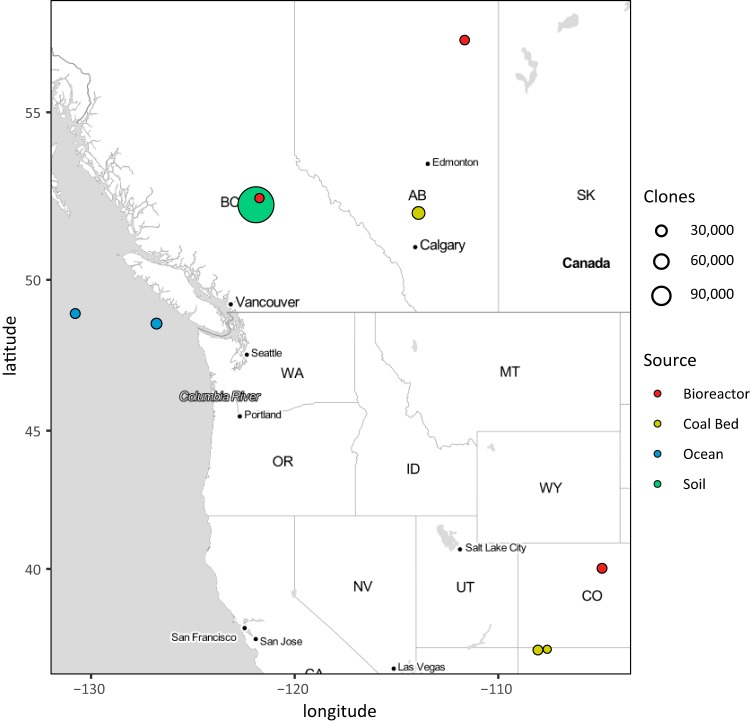
Fosmid library sampling locations. The size of fosmid libraries is indicated by the size of the bubbles, while the source type is indicated by the color.

Twenty-two fosmid libraries containing a total of 309,504 clones sourced from a variety of natural and engineered ecosystems were used in functional screens to identify active GH genes (Fig. 1; see also Table S1 in the supplemental material). Ocean water samples were collected along the line P transect in the northeastern subarctic Pacific (NESAP) Ocean at depths ranging from 10 to 2,000 m (18). Soil samples were collected from disturbed and undisturbed test plots in Williams Lake, British Columbia, at depths spanning organic and mineral soil horizons (19). Coal bed samples were collected from core cuttings or produced water (20). Bioreactor samples were collected from a passive mining bioremediation system (21), a methanogenic naphtha-degrading enrichment culture, or a methanogenic toluene-degrading enrichment culture (22). Each library typically contains between 10,000 and 25,000 clones harboring inserts between 32 and 47 kb in length ligated to the pCC1 copy control vector and hosted in Escherichia coli EPI300 cells. Averaging 1 gene per kb with an estimated genome size of 3 Mb, the combined set of libraries contains over 10^7^ open reading frames (ORFs) and around 4,000 genome equivalents. As the source environments varied widely in their biogeochemical and ecological dispositions, we anticipated that this diversity would potentiate the discovery of both known and novel GH genes through functional screening.

10.1128/mSystems.00082-19.6 TABLE S1 Library DNA source metadata. Download Table S1, DOCX file, 0.02 MB. Copyright © 2019 Armstrong et al. 2019 Armstrong et al. This content is distributed under the terms of the Creative Commons Attribution 4.0 International license.

### Functional screening.

The libraries were screened in a 384-well format with a fluorogenic substrate, 4-methylumbelliferyl β-cellobioside (MU-C), designed to detect cellulase, cellobiohydrolase, and β-glucosidase activity. This substrate releases the fluorophore 4-methylumbelliferone (MU), which we expected to be more sensitive than the previously employed 2,4-dinitrophenyl β-cellobioside (DNP-C), a chromogenic substrate which had been used to screen one-third (6,144 of 18,432) of the FOS62 clones (21), as fluorescence detection is inherently more sensitive. Although this substrate does not perfectly replicate the natural substrate encountered by these classes of enzymes, the fluorogenic reporter allows for sensitive detection of enzymes that have even mild activity. The use of this substrate was adapted to the screening paradigm employed by Mewis et al. (21), enabling 384-well-plate-based detection.

**FIG 2:**
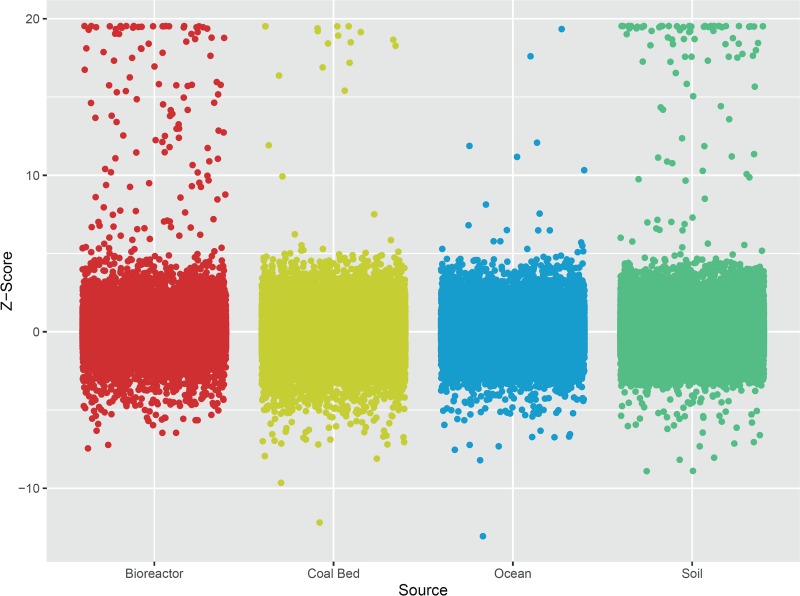
Functional screening of all libraries with MU-C. Robust Z-score values for fluorescence were calculated for each plate. Clones with a Z-score above 10 were chosen for further validation.

Functional screening recovered 164 verified hits with a robust Z-score above 10, indicating a hit rate of 1 in 1,887 clones, although recovery varied considerably between libraries (Fig. 2 and Table 1). The FOS62 library had the highest hit rate (1 in 239), followed by the toluene-degrading community (TolDC) library (1 in 794) and soil (average hit rate of 1 in 2,819). The coal bed and ocean libraries contained very few positive clones, with average hit rates of 1 in 7,795 and 1 in 17,920, respectively. The high hit rate for the FOS62 library is likely due to the enrichment of biomass-degrading genes as a result of the bioreactor being fed with partially degraded and composted cellulose and hemicellulose. As the FOS62 library had been previously screened with a different, chromogenic, substrate (DNP-C) (21), the performance of MU-C could be compared to this benchmark. For all FOS62 clones screened (*n* = 18,432), 90 colonies were determined to be hits with DNP-C (Z-score = 6), while 77 were uncovered with MU-C (Z-score = 10), with 35 of these clones recovered in both screens and a total of 132 unique clones identified. These two leaving groups appear to access somewhat different sets of enzymes, as 97 of the total 132 fosmids recovered from FOS62 (75%) were identified with only a single substrate. DNP-C is more reactive, as the pK_a_ of the 2,4-dinitrophenol aglycone (pK_a_ = 4.09) is substantially lower than that of MU (pK_a_ = 7.79), resulting in reduced activation energy for bond cleavage. The DNP-C probe, however, lacks the sensitivity of fluorogenic MU-C. A chemical activity probe bearing a fluorescent leaving group with low pK_a_ may afford a larger number of clones and offer an improved hit rate over either DNP-C or MU-C in isolation. Multiplex screening with a mixture of fluorescent probes provides another option for increased recovery in GH activity screens, as demonstrated recently on a fosmid library sourced from the beaver fecal microbiome (23).

**TABLE 1:** Functional screening hits identified with MU-C

Library	Source	No. of hits	No. of clones/hit
12010	Ocean	0	
12200	Ocean	1	7,680
12500	Ocean	1	7,680
40010	Ocean	0	
40500	Ocean	1	7,680
41000	Ocean	0	
41300	Ocean	0	
NO	Soil	12	896
NA	Soil	8	1,680
NB	Soil	5	1,997
NR	Soil	3	7,680
CO	Soil	4	4,128
CA	Soil	0	
CB	Soil	7	3,127
SCR	Soil	2	5,376
FOS62	Bioreactor	77	239
TolDC	Bioreactor	29	794
NapDC	Bioreactor	4	5,184
CG23A	Coal bed	2	4,800
CO182	Coal bed	4	5,760
CO183	Coal bed	0	
PWCG7	Coal bed	4	5,568
Total	Ocean	3	17,920
Total	Soil	41	2,819
Total	Bioreactor	110	566
Total	Coal bed	10	7,795
Total	All libraries	164	1,887

### High-throughput characterization of fosmids.

To gain further insight into the functional properties of active clones, including substrate preference, mechanism, pH dependence, and thermal stability, high-throughput characterization was performed by integrating the use of a Biomek FX workstation (Beckman Coulter) and plate-based assays without the need for enzyme subcloning and purification. It should be noted, however, that as more than one GH may be encoded and expressed on a given clone, this characterization may reflect the combined activities of more than one enzyme.

**FIG 3:**
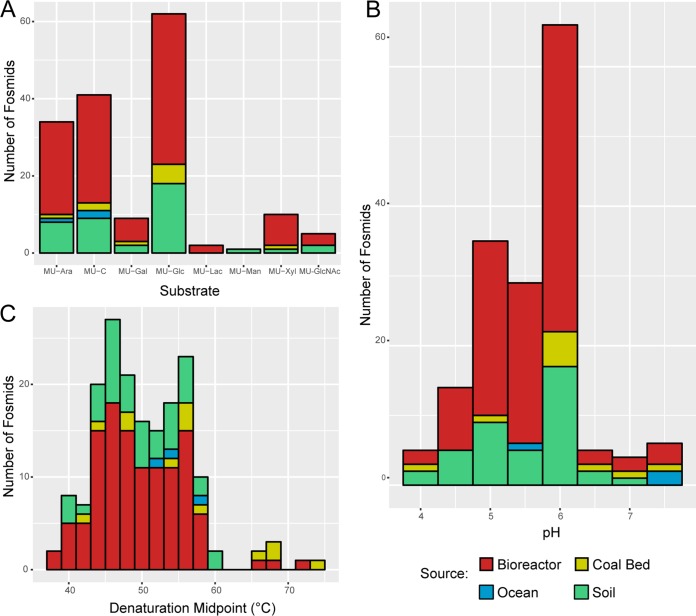
Fosmid substrate preference, pH optima, and thermal stability. (A) Each fosmid-containing clone was assayed against eight substrates: MU cellobioside (MU-C), MU lactoside (MU-Lac), MU β-d-glucopyranoside (MU-Glc), MU β-d-galactopyranoside (MU-Gal), MU β-d-xyloside (MU-Xyl), MU α-l-arabinofuranoside (MU-Ara), MU β-d-mannopyranoside (MU-Man), and MU *N*-acetyl-β-d-glucosaminide (MU-GlcNAc). Initial rates were determined using crude cell lysates to determine the optimal substrate for each clone. (B) Initial rates were used to determine the pH at which the fosmid-harboring clones best catalyzed the degradation of the optimal substrate. (C) Denaturation midpoints were determined for all clones by first preincubating the lysate over a range of temperatures and then assaying the clones with the optimal substrate to determine the initial rates of hydrolysis. Counts for each histogram are colored by library source.

**(i) Substrate preference.** Active clones were assayed against a panel of eight different glycosides bearing an MU leaving group. This panel of substrates consisted of cellobioside, lactoside, β-d-glucopyranoside, β-d-galactopyranoside, β-d-xylopyranoside, α-l-arabinofuranoside, β-d-mannopyranoside, and *N*-acetyl-β-d-glucosaminide. Many of these monosaccharides and disaccharides are present in the hemicellulosic and pectic fractions of wood. A majority of clones were most active against either the glucoside or cellobioside substrate; however, a substantial number of clones had higher activity against other substrates (Fig. 3A and Table S2). A total of 33 clones exhibited optimal activity against MU α-l-arabinofuranoside, and 10 clones exhibited optimal activity against MU β-d-xylopyranoside. These sugar monomers are essential components of hemicellulose (24), indicating that these fosmids may encode functions acting on both hemicellulose and glucosides. The presence of either multifunctional enzymes or multiple genes located in gene clusters such as polysaccharide utilization loci (PULs) is a likely explanation for the multiple activities seen, as described in the section on fosmid sequencing and gene annotation below.

10.1128/mSystems.00082-19.7 TABLE S2 Recovered fosmids, encoded activities, and genes. Download Table S2, PDF file, 0.3 MB. Copyright © 2019 Armstrong et al. 2019 Armstrong et al. This content is distributed under the terms of the Creative Commons Attribution 4.0 International license.

**(ii) Optimal pH determination.** To ascertain the optimal pH for each fosmid clone, assays were performed as indicated above, using the optimal substrate for that clone in a number of solutions buffered at pH values from 4.0 to 9.8. The average pH optimum was 5.7 ± 0.7, with the largest number of clones having an optimal pH of between 5 and 6 (130 of 164 clones) (Fig. 3B and Table S2). Of the clones with pH values of >7.5, a disproportionate number were derived from the ocean environment, likely reflecting the slightly alkaline pH of marine waters. A total of 5 clones were observed to have the lowest pH optima (CB006_04_L11, FOS62_34_K14, NO001_13_N07, PWCG7_49_G20, and TolDC_59_K14), being most active in pH 4 buffered solutions. No clear correlation between the sample pH and the optimal pH of the fosmid clone was observed. One possible explanation for this lack of correlation may be the intracellular use of a subset of these enzymes, causing the pH optima to be a reflection of intracellular pH rather than that of the environment.

**(iii) Thermal stability.** Further characterization was performed to determine the thermal stability of the activity seen for each clone. Assays were performed with the optimal substrate for each clone at its optimal pH, after preheating at a range of temperatures of between 37°C and 90°C. The resulting rates were used to determine the denaturation midpoint temperature (*T_m_*). The *T_m_* values determined spanned a range from 38°C to 74°C and had an average value of 50.6°C ± 6.6°C (Fig. 3C and Table S2). One noteworthy observation was that three of the four highest *T_m_* values determined were for fosmids from the PWCG7 library (PWCG7_33_K24, PWCG7_19_J20, and PWCG7_19_I21, with *T_m_* values of 69°C, 69°C, and 74°C, respectively). The PWCG7 library was sourced from coal bed-produced water that was at a temperature of 32.4°C, the highest temperature for any environment screened here (Table S1), consistent with this library producing the clones with the highest *T_m_*.

**(iv) Mechanism.** Glycosidases hydrolyze the glycosidic bond with one of two possible stereochemical outcomes: inversion or retention of anomeric stereochemistry. Inverting glycosidases carry out the reaction via an acid/base-catalyzed single-displacement reaction with an oxocarbenium ion-like transition state. In contrast, the vast majority of retaining glycosidases employ a two-step double-displacement mechanism involving the formation and hydrolysis of a covalent glycosyl enzyme intermediate. Our previously described development (25) of activated 2-deoxy-2-fluoroglycosides as reagents for the selective inhibition of retaining glycosidases via the formation of a stable 2-deoxy-2-fluoroglycosyl enzyme intermediate provides a convenient and high-throughput means for preliminary assessment of the reaction mechanism: if the 2-fluorosugar inhibits the enzyme, then it must be a retaining glycosidase. If not, then it is probably an inverting enzyme. Clones with the highest activity against MU-C, MU-Glc, MU-Gal, or MU-Xyl (122 in total) were assessed in this manner with their optimal 2-fluoroglycoside reagent, as described in Table S2. Of the clones interrogated, 58% were found to contain retaining glycosidases, with the remaining 42% most likely being inverters. Access to such information prior to fosmid sequencing would be valuable in the rapid selection of candidates for conversion to glycosynthases, since the strategy works much better with retaining enzymes.

### Fosmid sequencing and gene annotation.

Validated and characterized fosmids were then fully sequenced and assembled to reveal the genes present on each fosmid insert. Each clone was individually sequenced to completion using a multiplex approach on an Illumina MiSeq platform, generating a total of 5.7 Mbp of assembled data with an average fosmid insert size of 35,047 ± 4,852 bp. Comparison between sequences identified 115 nonredundant clones based on >95% similarity across >90% of the insert length. The redundant clones were most prevalent in the FOS62 and TolDC libraries (37 and 10 redundant clones, respectively), while there were no clones meeting the redundancy criteria identified within any of the soil libraries. This is consistent with the observed alpha diversity for bioreactor and soil milieus, with the highest diversity represented in organic and mineral soil horizons (22, 26, 27).

The taxonomic identity of the recovered fosmids was investigated through lowest-common-ancestor analysis using LCA* (28) and through identification of 16S rRNA genes on fosmids. Only 29 of the 164 fosmids could be reliably categorized at the phylum level or below using LCA*. Of these fosmids, 2 belong to the *Actinobacteria*, 5 belong to the *Proteobacteria*, and 22 belong to the phylum *Bacteroidetes* (Table S3). We were also able to identify two fosmids that carried 16S rRNA genes. Both TolDC_32_D22 and TolDC_55_H19 contained 16S rRNA genes, with both aligning with >98% identity to Sphaerochaeta associata and Sphaerochaeta globosa, respectively.

10.1128/mSystems.00082-19.8 TABLE S3 Fosmid taxonomy assigned by LCA*. Download Table S3, PDF file, 0.07 MB. Copyright © 2019 Armstrong et al. 2019 Armstrong et al. This content is distributed under the terms of the Creative Commons Attribution 4.0 International license.

### GH abundance.

**FIG 4:**
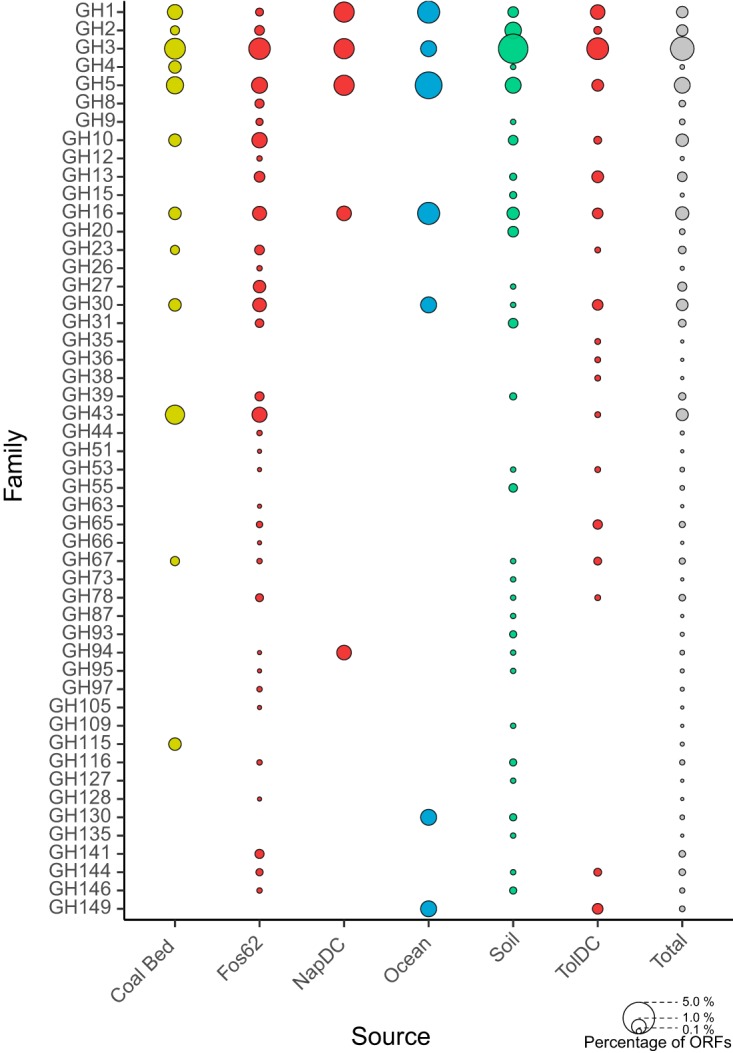
Predicted GH abundance on fosmid hits. Bubbles show the relative abundances of each GH family recovered from positive fosmid clones for each library source. The bioreactor results are shown for each library. Bubbles are colored by library.

Across all fosmids, 4,299 ORFs were predicted, an average of 26.2 ± 5.4 per fosmid (Table S2). These ORFs were queried against the CAZy database (29) with LAST (30) implemented in the MetaPathways pipeline (31), identifying 500 ORFs annotated as glycoside hydrolases (11.6% of all ORFs) (Fig. 4). The annotated GHs spanned 50 families, including all 7 families with β-glucosidase activity (GH1, -3, -5, -9, -30, -39, and -116) and 7 of 13 cellulase-containing families (GH5, -8, -9, -12, -26, -44, and -51). The two most abundant hydrolase families recovered from the fosmid inserts, GH3 and GH5 (2.7 and 1.2% of fosmid ORFs, respectively), are both retaining mechanism families, which is consistent with the large number of fosmids that contain retaining glucosidases, as determined by the above-described mechanistic investigations. The majority (44 of 46) of the identified GH5 enzymes belong to subfamilies with a known cellulase member (subfamilies 1, 2, 4, 25, 26, 37, 38, 39, 45, and 46) (Table S4). The final two subfamilies, GH5_36 and GH5_41, were both present on fosmids with another GH5 from a different subfamily. It is worth noting that GH5 subfamily 45 had until very recently lacked a characterized representative (32). As two of the identified fosmids that contain GH5_45 genes do not contain any other glucanase families, this supports the function of this subfamily on glucans.

10.1128/mSystems.00082-19.9 TABLE S4 Subfamilies of the identified GH5 and GH30 genes. Download Table S4, PDF file, 0.07 MB. Copyright © 2019 Armstrong et al. 2019 Armstrong et al. This content is distributed under the terms of the Creative Commons Attribution 4.0 International license.

An additional 5 hydrolase families accounted for more than 0.55% of all ORFs on the fosmid hits (GH16, GH43, GH10, GH30, and GH1, in order from most to least abundant), although not all of these families contain cellulases or β-glucosidases. The third most abundant family (GH16, with 0.8% of the predicted fosmid ORFs) is not annotated as containing either of these activities, but a portion of its characterized members cleave glucan polymers with mixed 1,3- and 1,4-linkages. All 30 of the fosmids annotated as containing a GH16 also contain either a GH3 or GH5. The large number of GH16s recovered is therefore likely due to their association with cellulases or β-glucosidases in clusters of genes that work together to degrade glucans.

GH43 enzymes have also not been described as cleaving β-glucans; rather, they are known to act on β-xylosides, α-l-arabinofuranosides, and β-galactans, which are key components of hemicellulose (33) and are often found in hemicellulose- and pectin-degrading loci (34, 35). As with the GH16 family, the high abundance of GH43 genes can be ascribed to their genomic colocalization with cellulases or β-glucosidases. Furthermore, the abundance of GH43s is likely the cause of the high percentage of hits with arabinosidase and xylosidase activities.

The distribution of predicted hydrolases on active clones was consistent across environments, barring a few exceptions. For example, very few GH1s were identified in the FOS62 library (5 genes; 0.25% of predicted ORFs) compared to all other environments (22 genes; 0.58% of predicted ORFs). The FOS62 library was also quite diverse in the range of endoglucanases recovered. Hydrolases belonging to the families GH8, GH12, GH44, and GH51 were recovered from only the FOS62 library.

**FIG 5:**
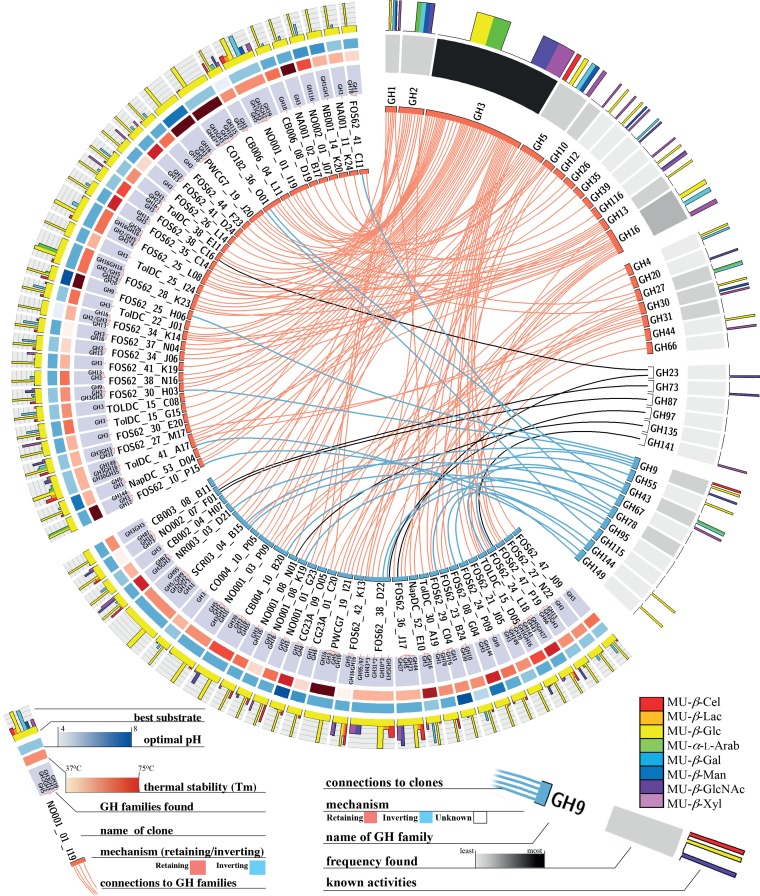
Circos plot for β-glucosidase clones. Clones with optimal activity on MU-Glc are shown with both activity and sequencing data. Substrate specificity, optimal substrate, optimal pH, thermal stability, and the results of mechanism testing are shown for each fosmid. The GH families found on each fosmid are also displayed. Individual clones are also linked to the GH families found on each clone. The families are labeled with the known activities, frequency of identification of the GH in this set of clones, and mechanism (if known). MU-β-Cel, 4-methylumbelliferyl β-cellobioside; MU-α-l-Arab, MU α-l-arabinofuranoside.

Sequencing of these fosmids also allowed us to investigate the patterns of gene presence and their correlation with observed activities (Fig. 5 and Fig. S1 to S3). Fosmids with the highest activities on monosaccharides all contained larger relative amounts of GH3s than the set most active on the cellobioside. Fosmids with the highest activity on cellobiosides, unsurprisingly, had a higher percentage of ORFs assigned to cellobiohydrolase-containing families GH5 and GH8. The set with the highest activity on MU-Glc had the highest diversity of GHs seen of any of the sets, which is likely a reflection of the greater number of fosmids within this group. The fosmids that were optimally active on MU-Xyl had a higher percentage of GH43s, a family containing β-xylosidases, than any other group of fosmids. This set of fosmids was also highly enriched in GH67 genes, which typically encode α-glucuronidases. This strongly suggests that gene cassettes on such fosmids are responsible for cleaving glucuronylxylan, hence the cooccurrence of glucuronidase and xylanase activities.

10.1128/mSystems.00082-19.2 FIG S1 Circos plot for cellobiohydrolase clones. Clones with optimal activity on MU-C are shown with both activity and sequencing data. Substrate specificity, optimal substrate, optimal pH, thermal stability, and the results of mechanism testing are shown for each fosmid. The GH families found on each fosmid are also displayed. Individual clones are also linked to the GH families found on each clone. The families are labeled with the known activities, frequency of identification of the GH in this set of clones, and mechanism (if known). Download FIG S1, EPS file, 1.7 MB. Copyright © 2019 Armstrong et al. 2019 Armstrong et al. This content is distributed under the terms of the Creative Commons Attribution 4.0 International license.

10.1128/mSystems.00082-19.3 FIG S2 Circos plot for α-l-arabinofuranosidase and β-galactosidase clones. Clones with optimal activity on MU-Gal or MU-Ara are shown with both activity and sequencing data. Substrate specificity, optimal substrate, optimal pH, thermal stability, and the results of mechanism testing are shown for each fosmid. The GH families found on each fosmid are also displayed. Individual clones are also linked to the GH families found on each clone. The families are labeled with the known activities, frequency of identification of the GH in this set of clones, and mechanism (if known). Download FIG S2, EPS file, 1.2 MB. Copyright © 2019 Armstrong et al. 2019 Armstrong et al. This content is distributed under the terms of the Creative Commons Attribution 4.0 International license.

10.1128/mSystems.00082-19.4 FIG S3 Circos plot for β-xylosidase clones. Clones with optimal activity on MU-Xyl are shown with both activity and sequencing data. Substrate specificity, optimal substrate, optimal pH, thermal stability, and the results of mechanism testing are shown for each fosmid. The GH families found on each fosmid are also displayed. Individual clones are also linked to the GH families found on each clone. The families are labeled with the known activities, frequency of identification of the GH in this set of clones, and mechanism (if known). Download FIG S3, EPS file, 0.6 MB. Copyright © 2019 Armstrong et al. 2019 Armstrong et al. This content is distributed under the terms of the Creative Commons Attribution 4.0 International license.

**FIG 6:**
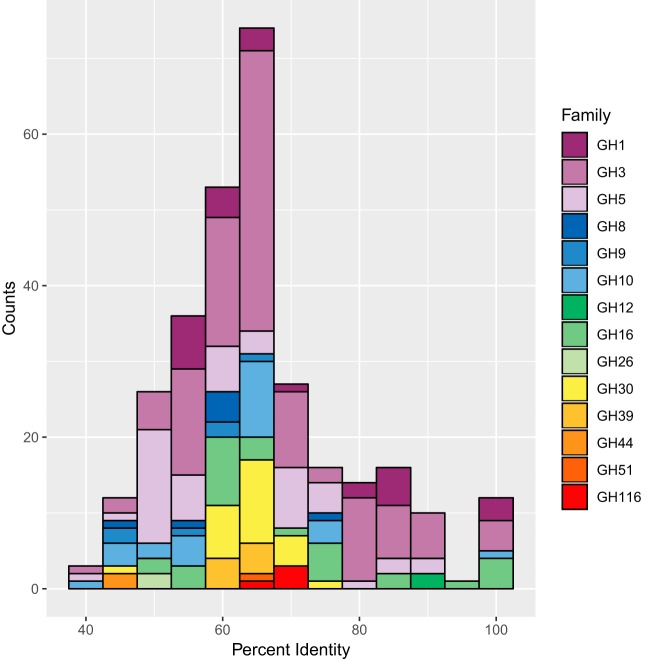
Percent identities of best BLAST hits to putative hydrolases. All putative hydrolase genes predicted to belong to β-glucosidase or cellulase families were queried against the BLAST nonredundant protein database.

To assess the distinctness of recovered glucanases, ORFs belonging to families containing β-glucosidase or cellulase activity were queried against the National Center for Biotechnology Information (NCBI) nonredundant protein database (accessed April 2017) using BLAST (36). The proteins recovered were quite distinct, with an average maximum identity of 65.5% ± 13.2% (Fig. 6). Of the 300 proteins queried, only 15 had a homologous protein with >90% identity. The lowest percent identity uncovered was that of the GH3 FOS62_47_P19_ORF8, which had a maximum identity of 40.5%. These results highlight the ability of functional metagenomic screening not only to find functional proteins but also to reveal a large number of previously unseen proteins.

### Presence of hydrolytic loci.

**FIG 7:**
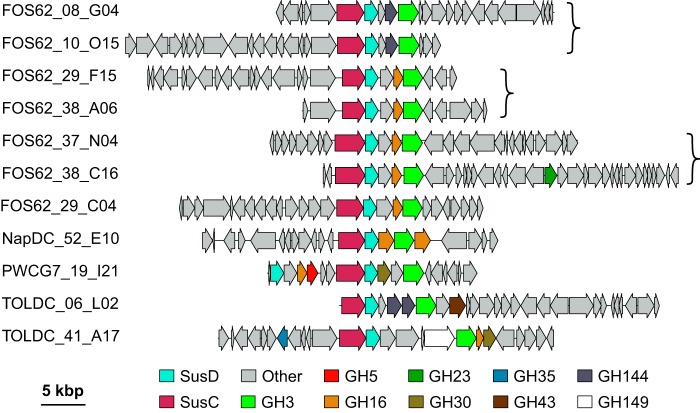
Gene organization of SusC/SusD-like protein-encoding fosmids. Putative glycoside hydrolases and SusC/SusD-like proteins are colored by family. ORFs not annotated as a glycoside hydrolase, SusC-like, or SusD-like are shown in gray. Fosmids identical to those shown here have been omitted for simplicity. Fosmids have been aligned to highlight synteny. Fosmids pairs or sets within brackets share >95% identity over the PUL region.

The wealth of fosmids encoding unexpected activities prompted an investigation of the PULs and gene clusters present on the fosmid hits. PULs have been reported in several cases to synergistically target complex plant polysaccharides, and their composition can give insight into the targeted polymers (34, 35, 37–40). A total of 11 fosmids were found to contain PULs, as indicated by the presence of the hallmark SusD/SusC-like gene pairing (Fig. 7). Several of the identified PULs shared nucleotide identity: FOS62_08_G04 and FOS62_10_O15 were identical at the nucleotide level over the PUL region, as were FOS62_29_F15 and FOS62_38_A06. Furthermore, fosmids FOS62_37_N04 and FOS62_38_C16 had 74% nucleotide identity over 97% of the identified PULs. Synteny was also seen for identified PULs, with the SusC/SusD pair being followed closely by a GH3 in all identified PULs and the frequent inclusion of either a GH16 or a GH144. This motif with a GH16 has been seen previously in a laminarin-degrading PUL from the marine bacterium Gramella forsetii KT0803 (37) and in the mixed-linkage glucan-degrading PUL from Bacteroides ovatus (41). This cooccurrence of GH3s and GH16s within the same gene cluster implies that many of these loci target noncellulosic glucans such as laminarin (a β-glucan containing 1,3- and 1,6-linkages) for degradation. The GH144 family, on the other hand, has thus far been shown to function only on β-1,2-glucans, suggesting that the GH144 containing PULs may also target this polymer.

**FIG 8:**
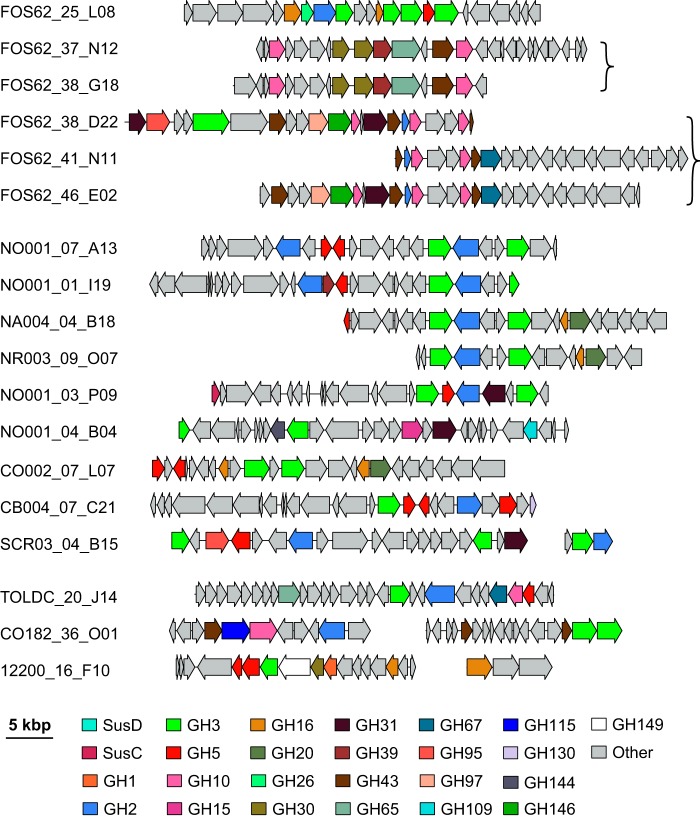
Gene organization of non-PUL fosmids containing more than 5 GH genes. Putative glycoside hydrolases proteins are colored by family. ORFs not annotated as a glycoside hydrolase are shown in gray. Fosmids identical to those shown here have been omitted for simplicity. Fosmids have been aligned to highlight synteny. Fosmids pairs or sets within brackets share >95% identity over their overlapping region.

Many of the fosmids that lack PULs contained clusters of multiple GH genes. More than 15% (25 of 164) of the fosmids contained 5 or more GH genes (Fig. 8). There was one set of two clones (FOS62_37_N12 and FOS62_38_G18) and an additional set of three clones (FOS62_38_D22, FOS62_41_N11, and FOS62_46_E02) with near-complete identity over an overlapping region. Additionally, a set of four clones (NO001_07_A13, NO001_01_I19, NA004_04_B18, and NR003_09_O07) share between 80 and 90% identity over a region containing a GH2 and two GH3 genes. Surprisingly, a number of clones were found with gene clusters that appeared to target xylans. These clones contained carbohydrate esterases, GH43s (which cleave xylosides and arabinosides), GH67s, and GH115s. Both GH67 and GH115 play a role in the removal of glucuronic acids from glucuronoxylan (42, 43). Specifically, clones CO182_36_O01, TOLDC_20_J14, FOS62_41_N11, and FOS62_46_E02 all contained either a GH67 or a GH115, while clones FOS62_37_N12, FOS62_38_D22, and FOS62_38_G18 harbored carbohydrate esterases predicted to target acetylations present on xylan. All of these potential xylan-targeting fosmids contain either a GH3, GH30, or GH10 enzyme. These are families with xylosidase or xylanase activity, and we posit that these are the proteins responsible for cleaving the MU-C screening substrate.

Several of the genes located on fosmids containing hydrolytic loci defied annotation. Furthermore, we were able to identify fosmids that contained carbohydrate-binding modules (CBMs) but lacked a catalytic domain (such as FOS62_41_N11_ORF0-CBM6 and CO182_36_O04_ORF9-CBM9). It is our hope that a detailed investigation of these genes could reveal new catalytic insights. Moreover, future characterization of the PULs and gene clusters has the potential to shed light on synergistic mechanisms of microbial organic matter conversion occurring within the sampled environments.

### Screening with modified glycosides and creation of a new glycosynthase.

**FIG 9:**
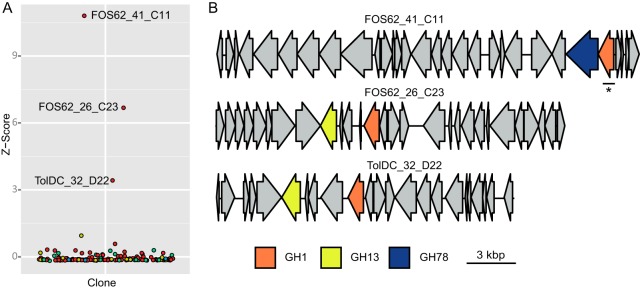
Screening of fosmid hits with a modified galactoside. (A) All 178 MU-C-active clones were screened with the azido-glucoside 6-N_3_-Gal MU. Z-score values for fluorescence were calculated for each plate. Individual clones are colored by source library as for Fig. 1. (B) Gene organization of fosmids with activity on 6-N_3_-Gal MU. Putative glycoside hydrolases are colored with the same scheme as in Fig. 5. ORFs not annotated as a glycoside hydrolase are shown in gray. The ORF selected for subcloning and further characterization (C11_GH1) is underlined.

The utility of the recovered GH-containing fosmids was demonstrated by identifying subsets of clones encoding activity on an unnatural substrate, 4-methylumbelliferyl 6-azido-6-deoxy-β-d-galactoside (6-N_3_-Gal MU), bearing an azido-functionality and subsequently transforming one of the identified enzymes into a glycosynthase capable of forming taggable disaccharides. The set of 164 fosmids was used to assay conversion of the 6-N_3_-Gal MU substrate, identifying three active clones with a robust Z-score of >3 (Fig. 9A). All three clones contained a gene for a GH1 enzyme, a family known to contain both β-glucosidases and β-galactosidases and thus likely to be the one responsible for the activity. However, each clone also carried one other GH gene: the FOS62_41_C11 fosmid contained a GH78 gene (rhamnosidase), while both FOS62_26_C23 and TolDC_32_D22 contained GH13 genes (typically α-glucosidase/α-amylase) (Fig. 9B).

As FOS62_41_C11 displayed the highest fluorescence, the GH1 from this fosmid was chosen for further characterization. The GH1-encoding gene was subcloned into a pET-28 expression vector and transformed into BL21(DE3) for expression. The expressed protein (C11-GH1) had suspected activity against 6-N_3_-Gal MU, with a specificity constant (*k*_cat_/*K_m_*) on the same order of magnitude as that of the unmodified glycoside (Table 2). Additionally, C11-GH1 cleaved 6-N_3_-Glc MU and the unmodified glucoside, with specificity constants an order of magnitude higher than for the corresponding galactosides.

**TABLE 2:** Kinetic constants determined for C11-GH1 on azido-modified and unmodified glycosides

Substrate	Mean *k*_cat_ (s^−1^) ± SD	Mean *K_m_* (mM) ± SD	Mean *k*_cat_/*K_m_* ratio (mM^−1^ s^−1^) ± SD
Gal MU	0.9 ± 0.2	0.05 ± 0.02	20 ± 9
6-N_3_-Gal MU	3.9 ± 0.5	0.10 ± 0.02	40 ± 10
Glc MU	63 ± 5	0.20 ± 0.03	320 ± 50
6-N_3_-Glc MU	14 ± 1	0.05 ± 0.01	280 ± 60

Based on this glycone specificity, we next sought to create a nucleophile variant of C11-GH1 and test for glycosynthase activity. The nucleophile residue (E354) was identified by homology to the GH1 Abg (44), and its codon was mutated to that encoding serine. This mutant gene was then transformed into an expression strain and expressed as described above for the cognate wild-type enzymes. Direct analysis of the cell lysate by thin-layer chromatography (TLC) and mass spectrometry showed that C11-GH1_E354S is capable of transferring galactose from α-d-galactosyl fluoride (α-F-Gal) onto pNP-Glc, *p*-nitrophenyl β-d-xylopyranoside (pNP-β-Xyl), and *p*-nitrophenyl α-d-xylopyranoside (pNP-α-Xyl). C11-GH1_E354S was also capable of using both 6-N_3_-α-F-Glc and 6-N_3_-α-F-Gal as glycosynthase donors with pNPG as an acceptor.

To further characterize the glycosynthase products generated by C11_E354S, we performed four such reactions on a small scale with 50 μmol of a donor sugar and 250 μmol of an acceptor, as shown in Table 3. Galactosylation of pNP-Glc resulted in both the 1,3- and 1,2-linked products (see Text S1 in the supplemental material for detailed nuclear magnetic resonance [NMR] assignment). GH1 glycosynthase production of 1,3-linked pNP galactosylglucoside has been previously observed for both Abg_E358A (9) and Bgl3_E383A (45); however, the 1,2-linked product has not been observed previously. Galactosylation of the xylosides resulted in 1,2-linkages when either pNP-α-Xyl or pNP-β-Xyl was used. The product containing the α-xyloside is particularly interesting, as this could be used as a model substrate for xyloglucan decorations. The galactosyl xylosides produced here have different linkages than those produced by either Abg_E358A (9) or Bgl3_E383A (45). Both these enzymes are able to catalyze the galactosylation of pNP-β-Xyl; however, in both cases, the major regiochemical outcome was the 1,3-linked product. We were also able to use C11_E354S to attach 6-azido-modified α-galactosyl fluoride. The glycosynthase reaction between 6-N_3_-α-F-Gal and pNP-Glc resulted in three separate products, with similar yields, as described in Table 3. The 1,2-, 1,3-, and 1,4-linked products were all observed, with the 1,3-glycoside being the major product. This is somewhat surprising, as the 1,4-linked product was not observed when the unmodified galactoside donor was used in a similar reaction. The regiochemical outcome is thus influenced by the presence of the 6-azido functional group. Taken together, these results demonstrate the ability of C11_E354S to glycosylate with azido-modified donors, and future research should focus on the scope of molecules that can act as competent acceptors. This may lead to additional products such as taggable inhibitors and activity-based probes.

**TABLE 3:** Disaccharides produced by the glycosynthase C11_GH1_E354S

Donor	Acceptor	Product	Yield (%)
α-F-Gal	pNP-Glc	Gal-(β-1,2)-Glc-β-pNP	15
		Gal-(β-1,3)-Glc-β-pNP	20

α-F-Gal	pNP-α-Xyl	Gal-(β-1,2)-Xyl-α-pNP	50

α-F-Gal	pNP-β-Xyl	Gal-(β-1,2)-Xyl-β-pNP	60

6-N_3_-α-F-Gal	pNP-Glc	6-N_3_-Gal-(β-1,4)-Glc-β-pNP	23
		6-N_3_-Gal-(β-1,2)-Glc-β-pNP	21
		6-N_3_-Gal-(β-1,3)-Glc-β-pNP	37

10.1128/mSystems.00082-19.1 TEXT S1 NMR data for reported compounds. Download Text S1, DOCX file, 0.1 MB. Copyright © 2019 Armstrong et al. 2019 Armstrong et al. This content is distributed under the terms of the Creative Commons Attribution 4.0 International license.

### Conclusions.

The coupling of liquid-based high-throughput functional screening, plate-based clone characterization, and fosmid sequencing and annotation has allowed us access to the cellobioside-degrading activities encoded in libraries sourced from a wide range of natural and engineered ecosystems resolving a genomic resource for downstream enzymology and synthetic biology applications in the biorefining space. This has revealed hundreds of glycoside hydrolases, many of which show low identity to any previously discovered gene, and provided validated functions for gene models predicted solely on the basis of metagenomic sequencing. The use of large-insert libraries also enables the recovery of gene cassettes with the potential to drive combinatorial biomass deconstruction, uncovering PULs and clusters of multiple GHs, many of which appear to target hemicelluloses. Furthermore, we have demonstrated the utility of this resource by first identifying clones that act on an unnatural glycoside and then using one of these clones as the basis for the design of a new glycosynthase. Further exploration of the utility of this suite of clones through interrogation with additional probes or substrates promises to expand our biocatalytic toolbox, with potential application to biomass deconstruction, the synthesis of glycans, and the development of activity-based probes.

## MATERIALS AND METHODS

### Sampling.

Soil samples were collected from the long-term soil productivity site (LTSP) at Skulow Lake, British Columbia, from the organic layer, mineral layer of eluviation, mineral transition layer, and mineral layer of accumulation at both undisturbed (libraries NO, NA, NB, and NR) and harvested (libraries CO, CA, CB, and SCR) sites (19). Water from the NESAP was collected at line P stations 4 and 12 in February 2010 at depths of between 10 and 2,000 m (18). Two coal bed samples were derived from cuttings of coal bed cores sourced from Rockyford standard (CO182) and basal (CO183) coal zones in Alberta. Another two samples (CG23A and PWCG7) were collected from coproduced water from coal bed methane well heads located in the San Juan Basin, New Mexico (20). Two bioreactor samples were derived from a methanogenic naphtha-degrading community (NapDC) (22) and a methanogenic toluene-degrading community (TolDC) (22, 46, 47). A homogenized core sample was collected from the final sampled bioreactor (FOS62) that was designed for remediation of metal-contaminated effluent and contained a mixture of limestone, quartz sand, and biosolids (including bacterial biomass and partially degraded and composted cellulose and hemicellulose) (21, 48). Details of environmental conditions and locations can be found in Table S1 in the supplemental material.

### Library creation.

Environmental DNA was isolated as described previously (18–22, 46, 47). Fosmid libraries were created from this purified DNA using the CopyControl fosmid library production kit with the pCC1FOS vector kit (Epicenter) (49). Briefly, the DNA was end repaired to create 5′-phosphorylated blunt ends and then subjected to pulsed-field gel electrophoresis to size select 35- to 60-kb DNA fragments. The DNA was recovered by gel extraction and ligated into the pCC1 vector. Linear concatemers of pCC1 and insert DNA were packaged into phage and transfected into phage-resistant EPI300 E. coli cells. The successfully transfected clones were recovered on Luria-Bertani (LB) agar plates containing chloramphenicol (12.5 μg/ml) and picked into 384-well plates, containing 100 μl of LB medium plus chloramphenicol (12.5 μg/ml) and 10% glycerol, with an automated colony-picking robot (Qpix2; Genetix). Clones were grown overnight at 37°C and then stored at −80°C. In total, fosmid library construction produced 309,504 individual clones from a diverse set of environments (Table S1). Libraries were replicated to create working copies of the original library, thereby reducing the freeze-thaw cycles and potential contamination of the original plates.

### Functional screening.

Screening was performed based on procedures described previously by Mewis et al. (21), with the only modification being the use of fluorogenic 4-methylumbelliferyl cellobioside (0.5 mM) as the screening substrate. Hits (Z-scores of >10) were validated by rescreening each clone in triplicate. These clones were rearrayed, using an automated colony-picking robot (Qpix2; Molecular Devices), into a 384-well plate containing 80 μl of LB medium, chloramphenicol (12.5 μg/ml), and 10% glycerol. This master plate was incubated overnight at 37°C and then stored at −80°C. Azide-containing substrates were synthesized by standard approaches, and their synthesis and characterization were reported previously (50). Screening of the master plate with MU 6-azido-6-deoxy-galactoside was performed under the same screening conditions.

### Fosmid-encoded activity characterization.

The master plate was inoculated into a 96-deep-well plate containing 800 μl of LB medium with chloramphenicol (12.5 μg/ml) and arabinose (100 μg/ml). After 18 h of growth at 37°C with shaking, cells were harvested by centrifugation at 3,200 × *g* for 20 min. The supernatant was decanted, cell pellets were resuspended in 100 μl of buffer (20 mM sodium acetate [NaOAc], 10 mM NaCl [pH 6.0]), and the optical density at 600 nm (OD_600_) was recorded. Next, 100 μl of 2× lysis buffer (20 mM NaOAc, 10 mM NaCl, 2% Triton X-100, 0.5 mg/ml lysozyme, cOmplete protease inhibitor-EDTA free [1 tablet per 50 ml; Sigma-Aldrich] [pH 6.0]) was added. The plate was incubated, with a glass bead in each well, on a Labnet Orbit P4 digital shaker at 600 rpm for 2 h at 25°C.

To initiate reactions, 20 μl of the lysate was added to a 96-well plate containing 100 μl of buffer and the substrate (20 mM NaOAc, 10 mM NaCl, 240 μM substrate [pH 6.0]). Reactions were performed with a Beckman Coulter Biomek FX workstation and run in triplicate at 20°C. Samples (10 μl) were taken after set intervals and quenched by addition to 100 μl of stop buffer (1 M glycine, pH 10.4). The fluorescence of quenched reactions was determined with a Beckman Coulter DTX-880 multimode detector (excitation wavelength [λ_ex_] of 365 nm and emission wavelength [λ_em_] of 465 nm). Initial rates, within 10% of substrate consumption, were used to quantify enzyme activity. To assess substrate preference, assays were performed with nine 4-methylumbelliferyl (MU) glycoside substrates: cellobioside, lactoside, β-d-glucopyranoside, β-d-galactopyranoside, β-d-xylopyranoside, α-l-arabinofuranoside, β-d-mannopyranoside, and *N*-acetyl-β-d-glucosaminide derivatives (Sigma-Aldrich).

Activity dependence on pH was determined using the above-described assay, with the optimal substrate and a set of citrate-phosphate buffers (50 mM sodium phosphate, 25 mM sodium citrate, 10 mM NaCl) with a pH of between 4 and 7.7, which was repeated in pH 7 to 9.8 glycyl-glycine buffers (20 mM) if necessary. Optimum pH was recorded as the pH at which the maximum velocity was observed. To assess thermal stability, lysate aliquots were preincubated for 10 min at a range of temperatures of between 37°C and 90°C using a 96-well MyCycler thermal cycler (Bio-Rad). Assays with the optimal substrate were then conducted as described above. Data were fitted to the van’t Hoff equation to deduce the denaturation midpoint temperature (*T_m_*).

The mechanism was determined by incubation of each clone with the activated 2-deoxy-2-fluoroglycoside corresponding to the parent glycoside substrate that showed the highest activity for that clone. Mechanistic tests were confined to those clones with the highest activity against cellobiosides, galactosides, glucosides, or xylosides. The substrates used for inactivation were the 2,4-dinitrophenyl derivatives of 2-deoxy-2-fluoro-cellobiose and 2-deoxy-2-fluoro-β-d-glucose as well as the recently described (51) 9H-(1,3-dichloro-9,9-dimethylacridin-7-one-2-yl) derivatives of 2-deoxy-2-fluoro-β-d-xylose and 2-deoxy-2-fluoro-β-d-galactose. After incubation for 1 h, the residual activity was assessed by adding the optimal substrate (240 μM) and monitoring fluorescence. Comparison of activity to that of a paired, uninhibited control revealed which clones were inactivated.

### Fosmid DNA isolation, sequencing, and assembly.

Fosmid DNA was isolated, sequenced, and assembled as previously described (52). Fosmid DNA was extracted from clones using the GeneJET plasmid miniprep kit (Thermo Fisher Scientific) according to the manufacturer’s instructions. Fosmid preparations were further treated with PlasmidSafe DNase (Epicenter) to degrade contaminating E. coli chromosomal DNA. Fosmid DNA concentrations were measured with a Quant-iT dsDNA HS assay kit (Invitrogen) using a Qubit fluorometer (Invitrogen). For full-fosmid sequencing, 2.4 ng of each fosmid was sent to the University of British Columbia Sequencing Centre (Vancouver, Canada). Each fosmid was individually barcoded and sequenced using the MiSeq system. All Illumina MiSeq raw sequence data were trimmed and assembled using a Python script available on GitHub (https://github.com/hallamlab/FabFos). Assemblies that yielded more than one contig were then scaffolded using minimus2 (53). Parameterized commands can be found in both documentations on the GitHub page and in the Python script itself.

### Annotation of fosmids.

Open reading frames (ORFs) were predicted using Prodigal (54) implemented in the MetaPathways pipeline (31). The 164 assembled fosmids yielded 4,299 ORFs of >180 nucleotides in length, which were annotated using LAST (30) implemented in the MetaPathways pipeline based on queries of the CAZy database (29) (retrieved on 25 March 2019) and the RefSeq protein database (retrieved on 18 January 2014). LCAStar (or LCA*) was used to assign taxonomy by minimizing entropy changes to the taxonomic distribution, i.e., the lowest common ancestor for each ORF on individual fosmid clones. The original LCA* codebase implemented by Hanson et al. (28) relied on files formatted specifically for the MetaPathways pipeline. A Python 3-compatible version (release v0.1; available at https://github.com/cmorganl/LCAStar) was forked and minimal to accept minimal inputs: a BLAST output table, the NCBI taxonomic tree, and a tabular file mapping NCBI numerical taxonomic identifiers to organism name and rank.

### Subcloning of C11_GH1 and mutagenesis.

C11_GH1 was inserted into a pET28 vector with a C-terminal His tag. Insert DNA was amplified via a PCR mixture containing deoxynucleoside triphosphates (dNTPs) (300 μM), forward and reverse primers (200 nM each) (Table 4), fosmid DNA (10 ng), Phusion polymerase (1 U; Thermo Fisher), and 1× reaction buffer. Cycling parameters were an initial denaturation step at 95°C (2 min) and 25 cycles of denaturation at 95°C (30 s), annealing at 72°C (30 s), and extension at 72°C (1 min). PCR products were digested with NdeI and XhoI (Thermo Fisher) and then ligated with dephosphorylated vector DNA that had been treated with the same endonucleases. The ligation mixture was transformed into DH5α cells, and plasmids were sequence verified and then transformed into BL21(DE3) cells for expression.

**TABLE 4:** Primers used in this study

Primer	Sequence
C11_GH1_F	GTACCATATGGTGGCTTTTTCGGATAAATTTTTGTG
C11_GH1_R	GTACCTCGAGTTACAGATTTTTTCCGTTCCTGCTG
C11_GH1_E354S_F	CCTGCCGCTTATTATTACCTCAAACGGGATGGCGGACAACGAC
C11_GH1_E354S_R	GTCGTTGTCCGCCATCCCGTTTGAGGTAATAATAAGCGGCAGG

The serine nucleophile mutant of C11_GH1 was generated using a modified QuikChange mutagenesis protocol (55). Primers used are detailed in Table 4. The DpnI (Thermo Fisher)-digested and purified PCR product (10 μl) was then used to transform DH5α cells, and plasmids were sequence verified and then transformed into BL21(DE3) cells for expression.

### Protein expression and purification.

C11_GH1 and the serine nucleophile variant C11_GH1_E354S were purified using a Ni-nitrilotriacetic acid (NTA) resin column. Medium (50 ml LBE-5052 [56] with 50 μg/liter of kanamycin) was inoculated with the expression host and incubated for 18 h at 37°C with shaking. Cultures were centrifuged (3,220 × *g* at 4°C for 20 min), the supernatant was removed, and cell pellets were stored at −80°C until purification. Next, 2.5 ml of lysis mix (1× BugBuster [Novagen], 20 mM HEPES, 300 mM NaCl, 50 mM imidazole [pH 7.0]) was used to resuspend the thawed cell pellets. This suspension was incubated at room temperature for 20 min, after which the lysate was clarified by centrifugation (3,220 × *g* at 4°C for 20 min) and loaded onto a column containing 1 ml of HisPur resin (Thermo Scientific). This mixture was washed with 20 ml of buffer A (20 mM HEPES, 300 mM NaCl, 50 mM imidazole [pH 7.0]), and the protein was eluted with 4 ml of buffer B (buffer A with 500 mM imidazole). Purified proteins were buffer exchanged into storage buffer (20 mM HEPES, 300 mM NaCl [pH 7.0]) with an Amicon 30,000-molecular-weight-cutoff (MWCO) filter column and stored at 4°C. The protein concentration was determined by the absorbance at 280 nm using a calculated extinction coefficient of 128,480 M^−1^ cm^−1^.

### Enzyme kinetics.

Kinetic parameters for C11_GH1 were determined using fluorogenic substrates. Assays were performed in 96-well plates containing the fluorogenic glycoside (0.5 μM to 1 mM), buffer (20 mM HEPES, 300 mM NaCl [pH 7.0]), and purified enzyme. Reactions were performed at 37°C, and fluorescence (λ_ex_ of 365 nm and λ_em_ of 450 nm) was monitored using a Synergy H1 plate reader (BioTek). The quantity of fluorophore generated was determined by means of a calibration curve of MU in an identical buffer system. Reactions were performed in triplicate. Kinetic parameters were calculated with the software program GraFit 7.0.

### Small-scale glycosynthase reactions.

Glycosynthase activity with donor sugars, α-d-galactosyl fluoride (α-F-Gal), 6-azido-6-deoxy-α-d-galactosyl fluoride (α-F-6-N_3_-Gal), or 6-azido-6-deoxy-α-d-glucosyl fluoride (α-F-6-N_3_-Glc), was evaluated on a 25-μl scale with 20 μM enzyme, 20 mM donor sugar, 20 mM the appropriate acceptor (*p*-nitrophenyl β-d-glucopyranoside [pNPG], *p*-nitrophenyl β-d-xylopyranoside [pNP-β-Xyl], or *p*-nitrophenyl α-d-xylopyranoside [pNP-α-Xyl] [sourced from Sigma-Aldrich]) in reaction buffer (100 mM HEPES, 100 mM NaCl [pH 7.0]). Reaction mixtures were incubated at 20°C for 18 h, after which they were monitored by thin-layer chromatography (TLC). TLC was performed on aluminum-backed sheets of silica gel 60F^254^ (E. Merck) with a thickness of 0.2 mm. The plates were visualized using UV light (254 nm) and/or by exposure to 10% ammonium molybdate (2 M in H_2_SO_4_), followed by charring. Reactions displaying product spots were sent for mass spectrometry analysis and selected for preparative-scale reactions.

### Preparative-scale reactions.

Preparative-scale reaction mixtures contained 1.5 μM C11_GH1_E354S, 2 mM donor sugar (α-F-Gal, α-F-6-N_3_-Gal, or α-F-6-N_3_-Glc), 10 mM the acceptor molecule (pNPG, pNP-α-Xyl, or pNP-β-Xyl), and reaction buffer. These reactions were performed on a 25-ml scale, and the mixtures were incubated at 25°C for 18 h with gentle agitation. Reactions were terminated by boiling for 10 min, and the mixtures were centrifuged and then lyophilized. Solid products were suspended in 500 μl of 5% (vol/vol) acetonitrile in water, passed through a Millipore Ultrafree MC centrifugal column (polyvinylidene difluoride [PVDF] [0.22 μm]), and then loaded onto a C_18_ column (Zorbax Eclipse XDB-C_18_, 9.4 mm by 250 mm; Agilent). A gradient of 5 to 10% (vol/vol) or 10 to 20% (vol/vol) acetonitrile in water was used to elute the product. The absorbance at 300 nm was monitored, and fractions corresponding to major products were pooled. Products were lyophilized and then prepared appropriately for mass spectrometry and NMR spectroscopy.

### Mass spectrometry and NMR spectroscopy of products.

^1^H, ^13^C, ^1^H-^1^H correlation spectroscopy (COSY), ^1^H-^1^H total correlation spectroscopy (TOCSY), ^1^H-^13^C heteronuclear single quantum coherence (HSQC) spectroscopy, and ^1^H-^13^C heteronuclear multiple bond correlation NMR spectra were collected on Bruker Avance 400-MHz and Bruker Avance III 600-MHz spectrometers at 25°C. All spectra were recorded using an internal deuterium lock and are referenced internally using the residual solvent peak. Chemical shifts are quoted in parts per million downfield of tetramethylsilane. Coupling constants (*J*) are given in hertz. Carbon NMR spectra were acquired with broadband proton decoupling and were recorded with distortionless enhancement by polarization transfer (DEPT). Mass spectra were measured on a Waters/Micromass LCT using electrospray ionization (ESI) with methanol as the solvent.

### Data availability.

Nucleotide sequences for the fosmids described have been deposited into GenBank (accession numbers MH105917 to MH106139).

10.1128/mSystems.00082-19.5 FIG S4 Circos plot for lactosidase, β-mannosidase, and *N*-acetyl-β-d-glucosaminide clones. Clones with optimal activity on MU-Lac, MU-Man, or MU-GlcNAc are shown with both activity and sequencing data. Substrate specificity, optimal substrate, optimal pH, thermal stability, and the results of mechanism testing are shown for each fosmid. The GH families found on each fosmid are also displayed. Individual clones are also linked to the GH families found on each clone. The families are labeled with the known activities, frequency of identification of the GH in this set of clones, and mechanism (if known). Download FIG S4, EPS file, 0.5 MB. Copyright © 2019 Armstrong et al. 2019 Armstrong et al. This content is distributed under the terms of the Creative Commons Attribution 4.0 International license.
