# Supplementary material for: High-Throughput Recovery and Characterization of Metagenome-Derived Glycoside Hydrolase-Containing Clones as a Resource for Biocatalyst Development
Source: mSystems. 2019 Jun 4;4(4):e00082-19. doi: 10.1128/mSystems.00082-19 (PMC6550366; doi:10.1128/mSystems.00082-19)
Supplement: TABLE S1 [file mSystems.00082-19-st001.docx]

Table S1: Fosmid Libraries Screened Within This Study

| Name | Category | Sample Type | Location | Ref. | Depth (m) | Temp. (°C) | pH | Clones |
| --- | --- | --- | --- | --- | --- | --- | --- | --- |
| 12010 | Ocean | Water from Station P12 | 48.969,-130.668 | Wright et al.[^1^](#_ENREF_1) | 10 | 8.4 | 7.8 | 7,680 |
| 12200 | Ocean | Water from Station P12 | 48.969,-130.668 | Wright et al.[^1^](#_ENREF_1) | 500 | 4.5 | 7.3 | 7,680 |
| 12500 | Ocean | Water from Station P12 | 48.969,-130.668 | Wright et al.[^1^](#_ENREF_1) | 2000 | 1.9 | 7.4 | 7,680 |
| 40010 | Ocean | Water from Station P4 | 48.650,-126.666 | Wright et al.[^1^](#_ENREF_1) | 10 | 9.9 | 7.8 | 7,680 |
| 40500 | Ocean | Water from Station P4 | 48.650,-126.666 | Wright et al.[^1^](#_ENREF_1) | 500 | 5.6 | 7.4 | 7,680 |
| 41000 | Ocean | Water from Station P4 | 48.650,-126.666 | Wright et al.[^1^](#_ENREF_1) | 1000 | 3.6 | 7.3 | 7,680 |
| 41300 | Ocean | Water from Station P4 | 48.650,-126.666 | Wright et al.[^1^](#_ENREF_1) | 1300 | 2.9 | 7.3 | 7,680 |
| NO | Soil | Natural; Organic horizon | 52.309,-121.902 | Hartmann et al.[^2^](#_ENREF_2) | 0 | 4.1 | 5 | 10,752 |
| NA | Soil | Natural; Mineral (eluviation) | 52.309,-121.902 | Hartmann et al.[^2^](#_ENREF_2) | 0.1 | 4.1 | 5.7 | 13,440 |
| NB | Soil | Natural; Mineral (transition) | 52.309,-121.902 | Hartmann et al.[^2^](#_ENREF_2) | 0.3 | 4.1 | 6 | 9,984 |
| NR | Soil | Natural; Mineral (accumulation) | 52.309,-121.902 | Hartmann et al.[^2^](#_ENREF_2) | 0.55 | 4.1 | 6.7 | 23,040 |
| CO | Soil | Clearcut; Organic horizon | 52.309,-121.902 | Hartmann et al.[^2^](#_ENREF_2) | 0 | 4.1 | 6 | 16,512 |
| CA23 | Soil | Clearcut; Mineral (eluviation) | 52.309,-121.902 | Hartmann et al.[^2^](#_ENREF_2) | 0.1 | 4.1 | 5.7 | 9,216 |
| CB | Soil | Clearcut; Mineral (transition) | 52.309,-121.902 | Hartmann et al.[^2^](#_ENREF_2) | 0.3 | 4.1 | 6.2 | 21,888 |
| SCR | Soil | Clearcut; Mineral (accumulation) | 52.309,-121.902 | Hartmann et al.[^2^](#_ENREF_2) | 0.55 | 4.1 | 6.7 | 10,752 |
| FOS62 | Bioreactor | Bioreactor core sample | 52.548, -121.633 | Mewis et al.[^3^](#_ENREF_3) | 0 | 18 | 6.9 | 18,432 |
| TolDC | Bioreactor | Toluene-degrading culture | 40.083, -104.8 | Tan et al.[^4^](#_ENREF_4) | 1.5 | 25 | 7.5 | 23,040 |
| NapDC | Bioreactor | Naphtha-degrading culture | 57.02, -111.55 | Tan et al.[^4^](#_ENREF_4) | 31 | 28 | 7.5 | 20,736 |
| CG23A | Coal Bed | Coal bed produced water | 37, -108 | An et al.[^5^](#_ENREF_5) | 300-500 | 32.1 | 7.9 | 9,600 |
| CO182 | Coal Bed | Coal bed cutting | 52.119, -113.78 | An et al.[^5^](#_ENREF_5) | 686 | 22 | N.D. | 23,040 |
| CO183 | Coal Bed | Coal bed cutting | 52.119, -113.78 | An et al.[^5^](#_ENREF_5) | 730 | 22 | N.D. | 23,040 |
| PWCG7 | Coal Bed | Coal bed produced water | 37, -108 | An et al.[^5^](#_ENREF_5) | 300-500 | 32.4 | 7.7 | 22,272 |
|  |  |  |  |  |  |  | **Total** | **309,504** |

N.D.: Not determined

References:

1. Wright, J. J.; Mewis, K.; Hanson, N. W.; Konwar, K. M.; Maas, K. R.; Hallam, S. J., *The ISME Journal* **2013,** 8 (2), 455-468.

2. Hartmann, M.; Lee, S.; Hallam, S. J.; Mohn, W. W., *Environmental Microbiology* **2009,** 11 (12), 3045-3062.

3. Mewis, K.; Armstrong, Z.; Song, Y. C.; Baldwin, S. A.; Withers, S. G.; Hallam, S. J., *Journal of biotechnology* **2013,** 167 (4), 462-71.

4. Tan, B.; Jane Fowler, S.; Laban, N. A.; Dong, X.; Sensen, C. W.; Foght, J.; Gieg, L. M., *The ISME Journal* **2015,** 9 (9), 2028-2045.

5. An, D.; Caffrey, S. M.; Soh, J.; Agrawal, A.; Brown, D.; Budwill, K.; Dong, X.; Dunfield, P. F.; Foght, J.; Gieg, L. M.; Hallam, S. J.; Hanson, N. W.; He, Z.; Jack, T. R.; Klassen, J.; Konwar, K. M.; Kuatsjah, E.; Li, C.; Larter, S.; Leopatra, V.; Nesbø, C. L.; Oldenburg, T.; Pagé, A. P.; Ramos-Padron, E.; Rochman, F. F.; Saidi-Mehrabad, A.; Sensen, C. W.; Sipahimalani, P.; Song, Y. C.; Wilson, S.; Wolbring, G.; Wong, M.-L.; Voordouw, G., *Environmental Science & Technology* **2013,** 47 (18), 10708-10717.
