# Supplementary material for: High-Throughput Recovery and Characterization of Metagenome-Derived Glycoside Hydrolase-Containing Clones as a Resource for Biocatalyst Development
Source: mSystems. 2019 Jun 4;4(4):e00082-19. doi: 10.1128/mSystems.00082-19 (PMC6550366; doi:10.1128/mSystems.00082-19)
Supplement: TABLE S2 [file mSystems.00082-19-st002.pdf]

Fosmid.Name	Source	Total_base_pairs	Predicted_ORFs	Redundant;_95%id90%_len	G+C(%)
12200_16_F10	Ocean	34693	20	No	39.6
12500_09_F02	Ocean	35450	34	No	37.7
40500_12_L11	Ocean	40578	30	No	48.91
CB002_04_H07	Soil	36180	20	No	54.25
CB003_08_B11	Soil	44051	25	No	55.49
CB004_07_C21	Soil	40929	23	No	53.63
CB004_10_B20	Soil	42146	35	No	53.04
CB005_08_O01	Soil	36021	33	No	57.19
CB006_04_L11	Soil	20694	14	No	56.37
CB006_08_D19	Soil	37925	33	No	57.07
CG23A_01_C20	Coal_Bed	36009	36	CG23A_01_C20	53.09
CG23A_09_O05	Coal_Bed	36005	36	CG23A_01_C20	53.08
CO002_07_L07	Soil	37582	22	No	54.15
CO003_01_D22	Soil	19386	12	No	48.06
CO004_05_B17	Soil	36391	24	No	52.12
CO004_10_P05	Soil	42348	32	No	54.92
CO182_11_I14	Coal_Bed	34526	30	No	63.47
CO182_24_J12	Coal_Bed	31857	26	CO183_09_B08	60.87
CO182_36_O01	Coal_Bed	42533	28	No	53.4
CO182_36_O04	Coal_Bed	37328	26	No	47.7
FOS62_08_C22	Bioreactor	32335	23	FOS62_08_G04	47.23
FOS62_08_D12	Bioreactor	34781	28	No	42.69
FOS62_08_G04	Bioreactor	32335	23	FOS62_08_G04	47.23
FOS62_10_O15	Bioreactor	36919	25	FOS62_10_O15	46.07
FOS62_10_P15	Bioreactor	36925	25	FOS62_10_O15	46.12
FOS62_21_B24	Bioreactor	33871	24	No	46.5
FOS62_21_D16	Bioreactor	29582	20	FOS62_43_C07	52.3
FOS62_21_J05	Bioreactor	37520	35	FOS62_21_J05	45.72
FOS62_22_C08	Bioreactor	36136	27	No	45.04
FOS62_23_B24	Bioreactor	38131	27	FOS62_43_C07	52.21
FOS62_23_J07	Bioreactor	38301	36	FOS62_23_J07	53.87
FOS62_24_J23	Bioreactor	31252	28	FOS62_23_J07	53.86
FOS62_24_L18	Bioreactor	32503	24	FOS62_40_E07	52.36
FOS62_24_P09	Bioreactor	31535	32	FOS62_25_H06	56.51
FOS62_25_H06	Bioreactor	34627	33	FOS62_25_H06	56.59
FOS62_25_L08	Bioreactor	37762	22	FOS62_25_L08	43.9
FOS62_25_O06	Bioreactor	34344	30	FOS62_25_O06	53.55
FOS62_26_C23	Bioreactor	30224	24	FOS62_26_C23	50.49
FOS62_26_C24	Bioreactor	30222	24	FOS62_26_C23	50.47
FOS62_26_K06	Bioreactor	39294	30	No	38.78
FOS62_26_K16	Bioreactor	36010	32	No	42.92
FOS62_26_L14	Bioreactor	32392	27	FOS62_38_N16	45.69
FOS62_27_M17	Bioreactor	32936	27	FOS62_46_D05	45.08
FOS62_27_N22	Bioreactor	39615	27	No	39.37
FOS62_27_P24	Bioreactor	38479	24	FOS62_27_P24	51.62
FOS62_28_A14	Bioreactor	38311	27	FOS62_40_E07	52.43

FOS62_28_K23	Bioreactor	37762	22	FOS62_25_L08	43.9
FOS62_29_C04	Bioreactor	35226	25	No	46.72
FOS62_29_F15	Bioreactor	35837	25	No	39.05
FOS62_30_E20	Bioreactor	29857	24	FOS62_38_N16	45.66
FOS62_30_H03	Bioreactor	34764	23	FOS62_30_H03	41.34
FOS62_30_J11	Bioreactor	33561	29	No	43.8
FOS62_30_L24	Bioreactor	37751	32	FOS62_30_L24	53.21
FOS62_30_N01	Bioreactor	34764	23	FOS62_30_H03	41.33
FOS62_34_D13	Bioreactor	23765	14	FOS62_38_G18	54.26
FOS62_34_J06	Bioreactor	33966	29	FOS62_38_N16	45.86
FOS62_34_K14	Bioreactor	30509	22	No	43.76
FOS62_35_C14	Bioreactor	33966	29	FOS62_38_N16	45.86
FOS62_36_J17	Bioreactor	36628	35	FOS62_23_J07	54.52
FOS62_36_K01	Bioreactor	34715	28	FOS62_30_L24	53.01
FOS62_37_C18	Bioreactor	23769	17	FOS62_43_C07	54.14
FOS62_37_N04	Bioreactor	35966	26	FOS62_37_N04	46.45
FOS62_37_N12	Bioreactor	35519	26	FOS62_37_N12	46.8
FOS62_38_A06	Bioreactor	21634	14	No	39.73
FOS62_38_C16	Bioreactor	44648	38	No	50.45
FOS62_38_D22	Bioreactor	37034	21	No	50.85
FOS62_38_G18	Bioreactor	27174	16	FOS62_38_G18	49.38
FOS62_38_N16	Bioreactor	35930	31	FOS62_38_N16	45.86
FOS62_40_E07	Bioreactor	34405	24	FOS62_40_E07	52.27
FOS62_40_G22	Bioreactor	35673	26	FOS62_37_N04	46.55
FOS62_41_A23	Bioreactor	35663	33	No	43.16
FOS62_41_C11	Bioreactor	36559	27	No	45.05
FOS62_41_D24	Bioreactor	32505	27	FOS62_38_N16	45.67
FOS62_41_I01	Bioreactor	34030	27	No	41.02
FOS62_41_K10	Bioreactor	27889	25	FOS62_30_L24	52.88
FOS62_41_K19	Bioreactor	27507	26	FOS62_21_J05	45.93
FOS62_41_L01	Bioreactor	32939	29	FOS62_25_O06	50.05
FOS62_41_N11	Bioreactor	31353	21	No	49.66
FOS62_42_D11	Bioreactor	33660	26	No	46.42
FOS62_42_K13	Bioreactor	33838	35	No	44.18
FOS62_43_C07	Bioreactor	36815	24	FOS62_43_C07	52.58
FOS62_43_F03	Bioreactor	30925	27	FOS62_38_N16	45.68
FOS62_43_J20	Bioreactor	24672	18	FOS62_43_C07	53.5
FOS62_43_J23	Bioreactor	25628	22	FOS62_30_L24	53.62
FOS62_43_O18	Bioreactor	37748	26	FOS62_27_P24	52.24
FOS62_44_A15	Bioreactor	35973	32	FOS62_30_L24	53.41
FOS62_44_E09	Bioreactor	29374	15	FOS62_27_P24	51.81
FOS62_44_F23	Bioreactor	31233	26	FOS62_38_N16	45.9
FOS62_44_J10	Bioreactor	38315	29	FOS62_44_J10	54.86
FOS62_46_D05	Bioreactor	33178	26	FOS62_46_D05	44.97
FOS62_46_E02	Bioreactor	40402	26	No	50.18
FOS62_46_L17	Bioreactor	36019	24	FOS62_43_C07	52.41
FOS62_47_B05	Bioreactor	30279	22	FOS62_44_J10	55.19

FOS62_47_F04	Bioreactor	28914	22	FOS62_43_C07	51.31
FOS62_47_H05	Bioreactor	31186	25	FOS62_46_D05	45.06
FOS62_47_J09	Bioreactor	38130	33	FOS62_21_J05	45.36
FOS62_47_P19	Bioreactor	31777	21	No	44.91
NA001_01_P12	Soil	41983	33	No	56.03
NA001_02_B17	Soil	35706	32	No	56.54
NA001_07_E13	Soil	31618	21	No	58.67
NA001_07_F24	Soil	37450	27	No	53.13
NA001_11_K24	Soil	41912	34	No	58.13
NA001_16_B03	Soil	22883	15	No	54.13
NA002_01_B04	Soil	41997	21	No	54.26
NA004_04_B18	Soil	34226	21	No	53.64
NapDC_20_D21	Bioreactor	41493	38	No	57.09
NapDC_21_E17	Bioreactor	26654	16	No	48.42
NapDC_52_E10	Bioreactor	35194	20	No	41.68
NapDC_53_D04	Bioreactor	36106	27	No	60.02
NB001_03_I24	Soil	37863	25	No	50.94
NB001_12_A01	Soil	37154	25	No	56.57
NB001_13_B14	Soil	33385	26	No	59.49
NB001_14_K20	Soil	33403	31	No	66.96
NB001_23_D20	Soil	35294	24	No	57.17
NO001_01_G23	Soil	41645	24	No	54.46
NO001_01_I19	Soil	39418	23	No	54.36
NO001_03_P09	Soil	35666	20	No	52.38
NO001_04_B04	Soil	41349	27	No	52.29
NO001_07_A13	Soil	37890	21	No	55.03
NO001_08_K19	Soil	37244	19	No	55.18
NO001_08_N01	Soil	45530	36	No	55.54
NO001_10_L12	Soil	42565	30	No	52.93
NO001_13_N07	Soil	22704	19	No	69.01
NO002_01_J07	Soil	40649	32	No	55.68
NO002_04_P09	Soil	43906	36	No	58.66
NO002_07_F01	Soil	36126	10	No	54.52
NR003_03_D21	Soil	42029	27	No	54.91
NR003_09_O07	Soil	36434	20	No	52.87
NR003_36_K13	Soil	36579	23	No	54.83
PWCG7_19_I21	Coal_Bed	33413	22	PWCG7_19_I21	41.28
PWCG7_19_J20	Coal_Bed	33397	22	PWCG7_19_I21	41.28
PWCG7_33_K24	Coal_Bed	34323	26	No	49.37
PWCG7_49_G20	Coal_Bed	38233	33	No	48.42
SCR03_01_L21	Soil	26582	16	No	53.33
SCR03_04_B15	Soil	45903	23	No	52.6
TolDC_06_L02	Bioreactor	36826	26	No	49.79
TolDC_08_I17	Bioreactor	44440	34	TOLDC_08_I17	53.45
TolDC_10_A11	Bioreactor	33947	27	TOLDC_08_I17	52.86
TolDC_15_C08	Bioreactor	35030	28	TOLDC_15_C08	52.68
TolDC_15_D05	Bioreactor	32791	27	TolDC_39_M03	50.94

TolDC_15_E19	Bioreactor	32947	27	No	53.33
TolDC_15_G15	Bioreactor	35021	29	TOLDC_15_C08	52.68
TolDC_20_J14	Bioreactor	38367	29	TOLDC_20_J14	55.67
TolDC_22_A01	Bioreactor	35017	32	No	49.97
TolDC_22_J01	Bioreactor	36226	28	TOLDC_08_I17	54.04
TolDC_25_I24	Bioreactor	32298	27	No	45.17
TolDC_30_A19	Bioreactor	30289	25	No	46.37
TolDC_30_J10	Bioreactor	41647	35	No	56.18
TolDC_31_E21	Bioreactor	38338	29	TOLDC_20_J14	55.68
TolDC_32_D22	Bioreactor	34208	27	No	48.75
TolDC_35_I03	Bioreactor	36951	29	No	46.32
TolDC_38_E11	Bioreactor	32602	25	TOLDC_08_I17	53.7
TolDC_39_M03	Bioreactor	40430	29	TolDC_39_M03	50.19
TolDC_41_A17	Bioreactor	38832	25	No	50.44
TolDC_46_B16	Bioreactor	32020	26	TOLDC_08_I17	53.63
TolDC_50_B06	Bioreactor	34869	27	TOLDC_08_I17	53.32
TolDC_50_P08	Bioreactor	34149	29	TOLDC_15_C08	52.43
TolDC_55_H19	Bioreactor	31460	21	No	50.3
TolDC_56_H11	Bioreactor	40331	31	No	50.94
TolDC_56_L15	Bioreactor	31169	26	No	52.82
TolDC_59_E21	Bioreactor	31860	26	TolDC_59_J06	49.69
TolDC_59_J01	Bioreactor	34372	31	No	48.46
TolDC_59_J06	Bioreactor	37327	31	TolDC_59_J06	49.87
TolDC_59_K14	Bioreactor	37802	32	No	49

MU-b-Cel	MU-b-Lac	MU-b-Glc	MU-a-L-Ara	MU-b-Gal	MU-b-Man	MU-b-GlcN	MU-b-Xyl	Optimal_Su
100	67	3	9	6	0	0	21	MU-C
100	71	0	1	1	0	0	0	MU-C
55	14	17	100	0	11	1	19	MU-Ara
5	3	100	0	35	0	1	0	MU-Glu
11	3	100	3	2	0	0	1	MU-Glu
2	0	15	8	3	100	18	3	MU-Man
8	0	100	5	1	1	58	16	MU-Glu
9	3	3	6	1	0	100	2	MU_GlcNA
50	8	100	26	28	0	56	25	MU-Glu
2	2	100	3	18	0	2	5	MU-Glu
21	0	100	3	0	1	4	1	MU-Glu
27	18	100	11	12	0	0	0	MU-Glu
100	86	0	0	7	0	0	0	MU-C
100	68	1	1	0	0	0	1	MU-C
2	2	26	2	53	0	0	100	MU-Xyl
12	0	100	7	30	1	14	7	MU-Glu
16	1	51	100	5	0	0	12	MU-Ara
1	1	6	4	100	0	0	1	MU-Gal
12	0	100	3	7	0	6	2	MU-Glu
100	61	46	0	80	7	0	12	MU-C
2	1	3	0	2	0	6	100	MU-Xyl
100	93	7	6	12	1	0	64	MU-C
14	18	100	9	4	0	1	0	MU-Glu
0	0	11	100	0	0	0	0	MU-Ara
10	7	100	97	0	0	2	2	MU-Glu
3	2	6	6	100	0	1	1	MU-Gal
5	3	2	100	28	0	16	0	MU-Ara
11	0	100	3	6	0	7	2	MU-Glu
18	0	53	100	71	0	0	36	MU-Ara
19	0	100	5	46	0	9	9	MU-Glu
100	88	0	0	0	0	0	0	MU-C
100	89	0	0	0	0	0	0	MU-C
50	31	100	1	4	0	19	22	MU-Glu
22	4	100	0	1	0	1	16	MU-Glu
38	9	100	25	10	0	7	10	MU-Glu
18	0	100	1	33	0	0	10	MU-Glu
2	2	3	28	100	1	9	0	MU-Gal
15	5	33	17	100	2	1	1	MU-Gal
14	1	74	31	14	0	100	62	MU_GlcNA
100	55	5	0	2	1	1	0	MU-C
100	46	45	0	1	2	0	1	MU-C
8	0	100	0	10	0	0	65	MU-Glu
44	0	100	47	38	0	61	21	MU-Glu
12	13	100	9	32	0	8	0	MU-Glu
100	95	0	0	0	0	0	0	MU-C
100	90	0	0	0	0	0	0	MU-C

17	1	100	88	2	0	1	1	MU-Glu
5	2	100	2	15	0	1	0	MU-Glu
8	0	17	100	0	0	0	0	MU-Ara
6	0	100	0	13	0	0	85	MU-Glu
30	0	100	16	23	0	47	21	MU-Glu
100	73	1	2	13	1	0	0	MU-C
100	78	5	0	0	2	0	2	MU-C
2	0	7	1	1	0	100	1	MU_GlcNA
100	48	6	0	7	6	0	3	MU-C
19	0	100	2	65	0	5	2	MU-Glu
4	0	100	4	2	0	7	1	MU-Glu
11	1	100	2	28	0	1	1	MU-Glu
73	29	100	0	0	0	90	67	MU-Glu
100	89	1	6	0	0	0	0	MU-C
18	10	3	100	75	3	25	1	MU-Ara
21	0	100	2	68	0	17	3	MU-Glu
100	43	5	0	2	6	2	3	MU-C
3	0	7	100	0	0	0	0	MU-Ara
5	5	100	3	20	0	0	79	MU-Glu
54	23	100	0	1	0	46	46	MU-Glu
100	47	11	0	0	14	0	2	MU-C
11	0	100	1	26	0	3	0	MU-Glu
100	82	1	0	0	0	0	0	MU-C
0	0	3	100	0	0	0	0	MU-Ara
1	0	10	100	1	0	0	0	MU-Ara
0	0	100	0	0	0	0	0	MU-Glc
13	2	100	1	28	0	0	34	MU-Glu
45	100	1	0	1	0	0	0	MU-Lac
100	99	0	0	0	0	0	0	MU-C
4	0	100	0	12	0	0	42	MU-Glu
66	100	22	0	81	3	1	11	MU-Lac
11	2	1	0	1	1	3	100	MU-Xyl
100	65	0	0	0	0	0	0	MU-C
95	58	100	0	1	0	63	61	MU-Glu
100	63	10	0	11	6	0	1	MU-C
32	0	19	0	3	0	3	100	MU-Xyl
100	39	2	2	2	2	7	2	MU-C
100	92	0	0	3	0	0	0	MU-C
100	83	0	0	0	0	0	0	MU-C
100	87	0	11	5	0	0	0	MU-C
100	81	0	0	0	0	0	0	MU-C
13	0	100	1	30	0	0	5	MU-Glu
51	22	87	15	100	0	4	0	MU-Gal
0	2	3	5	100	0	0	0	MU-Gal
8	2	1	0	6	1	3	100	MU-Xyl
100	32	11	37	2	10	24	7	MU-C
100	76	9	0	2	0	0	3	MU-C

100	50	2	0	1	2	0	1	MU-C
2	1	87	1	100	0	0	6	MU-Gal
17	0	100	2	54	0	2	1	MU-Glu
11	0	100	19	6	0	63	6	MU-Glu
1	1	11	100	1	0	0	1	MU-Ara
2	3	100	1	26	0	0	0	MU-Glu
100	67	6	0	1	0	0	0	MU-C
0	0	0	100	0	0	0	0	MU-Ara
4	1	100	0	22	0	0	1	MU-Glu
12	7	39	100	10	0	25	12	MU-Ara
28	7	85	100	49	0	70	43	MU-Ara
100	49	21	0	2	19	0	4	MU-C
100	10	6	0	1	1	0	2	MU-C
7	3	26	100	6	0	20	2	MU-Ara
14	2	100	1	91	0	0	34	MU-Glu
18	11	100	7	17	0	10	10	MU-Glu
5	3	7	100	2	0	1	1	MU-Ara
100	55	75	12	35	0	0	13	MU-C
100	48	42	6	32	1	0	13	MU-C
1	1	100	2	10	0	0	2	MU-Glu
4	2	8	100	1	0	0	5	MU-Ara
82	0	100	0	8	0	0	53	MU-Glu
38	32	100	0	94	40	3	40	MU-Glu
16	0	100	0	3	1	4	11	MU-Glu
0	0	1	3	100	0	0	0	MU-Gal
39	18	50	100	1	38	2	32	MU-Ara
36	12	100	0	2	3	25	15	MU-Glu
42	0	100	0	3	0	0	23	MU-Glu
77	58	10	1	6	0	100	3	MU_GlcNA
100	73	1	2	17	0	5	2	MU-C
0	1	100	0	54	0	0	0	MU-Glu
4	0	13	100	0	0	6	10	MU-Ara
37	24	100	6	9	0	51	24	MU-Glu
67	16	100	0	17	0	96	73	MU-Glu
100	55	36	0	0	0	14	19	MU-C
100	54	17	0	1	0	0	0	MU-C
2	1	100	1	2	0	1	1	MU-Glu
2	2	100	1	7	0	0	0	MU-Glu
37	6	5	0	-3	6	2	100	MU-Xyl
100	66	0	1	0	0	1	0	MU-C
6	5	4	4	100	0	0	0	MU-Gal
6	0	100	0	1	3	1	4	MU-Glu
7	0	47	100	0	0	0	2	MU-Ara
2	0	12	100	0	0	0	2	MU-Ara
12	0	57	100	1	0	1	8	MU-Ara
21	1	100	1	70	0	0	14	MU-Glu
5	-1	100	0	12	14	46	9	MU-Glu

9	0	24	100	0	0	0	3	MU-Ara
24	2	100	1	59	0	0	11	MU-Glu
31	15	0	0	0	0	1	100	MU-Xyl
0	1	11	100	1	0	1	0	MU-Ara
23	0	100	1	58	0	0	15	MU-Glu
0	0	100	0	5	0	0	7	MU-Glu
49	12	100	9	0	6	0	4	MU-Glu
4	0	31	100	1	0	0	4	MU-Ara
24	11	7	0	0	1	1	100	MU-Xyl
2	1	2	100	0	0	0	0	MU-Ara
19	0	3	100	26	1	21	0	MU-Ara
18	1	100	3	62	0	0	12	MU-Glu
0	0	43	100	0	0	17	0	MU-Ara
53	29	100	26	15	0	34	20	MU-Glu
20	1	81	100	2	0	0	15	MU-Ara
16	4	23	100	2	0	0	13	MU-Ara
18	9	10	2	4	0	0	100	MU-Xyl
73	33	66	0	27	0	2	100	MU-Xyl
16	0	72	2	0	0	100	1	MU_GlcNA
9	8	19	100	53	0	23	0	MU-Ara
100	64	96	3	46	0	4	4	MU-C
9	3	9	100	3	0	0	4	MU-Ara
8	2	56	100	1	0	0	15	MU-Ara
100	62	0	1	2	0	0	0	MU-C

pH_optimu	Tm_(Celsiu	Inhibition_	Number_of_Hydrolase	GH1	GH2	GH3	GH4
7.6	54	Yes	8	1	0	1	0
7.6	58	Yes	2	1	0	0	0
5.63	53	N/A	1	0	0	0	0
5	44	No	1	0	0	1	0
6	48	No	2	0	0	2	0
4.5	54	N/A	6	0	1	1	0
6	53	No	4	0	0	1	0
6	46	N/A	2	0	0	0	0
4	48	Yes	2	0	0	1	0
5.5	50	Yes	1	0	0	0	0
6.64	42	No	2	1	0	0	1
7.6	56	No	2	1	0	0	1
6	55	Yes	7	0	0	2	0
7.15	59	Yes	3	1	0	1	0
4.5	40	No	2	0	0	1	0
5.14	44	No	1	0	0	1	0
6	44	N/A	1	0	0	0	0
6	56	Yes	2	0	0	0	0
5.14	67	Yes	8	0	1	2	0
6	54	No	3	0	0	2	0
5.14	52	Yes	2	0	0	1	0
6	56	Yes	3	0	0	0	0
7.2	56	No	2	0	0	1	0
6.64	50	N/A	3	0	0	1	0
6	72	Yes	3	0	0	1	0
5.63	58	Yes	3	0	0	1	0
6	46	N/A	1	0	0	0	0
5.14	48	No	1	0	0	1	0
6	41	N/A	4	1	0	0	0
4.5	47	No	2	0	0	0	0
6	56	Yes	4	0	0	0	0
6	55	Yes	3	0	0	0	0
6	54	No	4	0	0	0	0
6	51	No	1	0	0	0	0
7.6	68	Yes	1	0	0	0	0
5.63	49	Yes	9	0	1	4	0
6	49	Yes	2	0	0	0	0
5.14	46	Yes	3	1	0	0	0
4.5	57	N/A	3	1	0	0	0
6	52	Yes	1	0	0	0	0
6	54	Yes	2	0	0	0	0
6	41	Yes	1	0	0	1	0
5.14	45	Yes	3	0	0	2	0
5	47	No	1	0	0	1	0
6	56	Yes	5	0	0	0	0
6	55	Yes	5	0	0	0	0

5.63	50	Yes	9	0	1	4	0
7.7	46	No	3	0	0	1	0
4.5	59	N/A	2	0	0	1	0
6	43	Yes	1	0	0	1	0
5.14	46	Yes	4	0	1	2	0
6	51	Yes	2	0	0	0	0
6	54	Yes	4	0	0	0	0
5.14	51	N/A	4	0	1	2	0
5.63	46	No	7	0	0	0	0
5.14	41	Yes	2	0	0	1	0
4	45	Yes	4	0	1	1	0
6	56	Yes	2	0	0	1	0
6	44	No	4	0	0	0	0
6	56	Yes	4	0	0	0	0
5.63	44	N/A	4	0	0	0	0
6	39	Yes	2	0	0	1	0
5.14	52	No	7	0	0	0	0
6	67	N/A	4	0	0	1	0
5	52	Yes	3	0	0	1	0
6	44	No	13	0	1	1	0
5.14	51	No	7	0	0	0	0
5.63	50	Yes	2	0	0	1	0
6	52	Yes	5	0	0	0	0
6.64	58	N/A	2	0	0	1	0
5.14	52	N/A	1	1	0	0	0
6	42	Yes	2	1	0	0	0
5.63	55	Yes	2	0	0	1	0
5.63	47	N/A	3	0	0	0	0
6	57	Yes	2	0	0	0	0
6	43	Yes	1	0	0	1	0
6	53	N/A	2	0	0	0	0
6	45	No	6	0	1	0	0
6	53	Yes	4	0	0	0	0
5.63	38	No	3	0	0	0	0
5.14	50	No	4	0	0	0	0
6	46	Yes	1	0	0	1	0
6	48	No	4	0	0	0	0
5.63	57	Yes	2	0	0	0	0
5.63	46	Yes	6	0	0	0	0
6	55	Yes	3	0	0	0	0
6	54	Yes	5	0	0	0	0
5.63	51	Yes	1	0	0	1	0
5.5	47	No	2	0	0	0	0
7.7	44	Yes	3	0	0	2	0
6	41	No	11	0	1	0	0
5.63	48	No	4	0	0	0	0
5.5	50	Yes	2	0	0	0	0

5.63	50	No	2	0	0	0	0
5	41	No	3	0	0	2	0
5.14	49	No	1	0	0	1	0
5.14	48	No	4	0	0	2	0
5.14	48	N/A	2	1	0	0	1
5.5	60	Yes	1	0	0	1	0
5	50	Yes	1	0	0	1	0
5.14	46	N/A	2	0	0	1	0
5	45	Yes	1	0	0	1	0
6	46	N/A	4	0	0	1	0
6	43	N/A	5	0	2	2	0
6	56	No	6	0	1	2	0
4.5	54	Yes	1	0	0	0	0
6	45	N/A	3	1	0	1	0
5	59	No	2	0	0	1	0
7.15	45	Yes	2	1	0	0	0
4.5	55	N/A	1	0	0	1	0
4.5	57	Yes	1	0	0	0	0
4.5	58	No	3	1	0	0	0
6.6	45	Yes	2	2	0	0	0
6	51	N/A	5	0	0	1	0
6	50	No	2	0	1	1	0
5	47	Yes	6	0	2	2	0
5.63	47	No	5	0	1	2	0
5.63	56	Yes	6	0	0	2	0
6	60	N/A	6	0	2	2	0
6	41	No	2	0	0	1	0
6	49	No	2	0	0	1	0
5	46	N/A	4	0	0	1	0
4	53	No	3	0	0	0	0
6.6	53	Yes	1	0	0	0	0
6	46	N/A	1	0	0	0	0
6	40	No	3	0	0	0	0
5.63	56	No	3	0	0	2	0
6	57	No	5	0	1	2	0
5	46	Yes	1	0	0	1	0
6	74	No	4	0	0	1	0
7.2	69	Yes	4	0	0	1	0
6	69	No	4	0	0	0	0
4	59	No	1	1	0	0	0
6	54	Yes	2	0	0	2	0
6	47	No	9	0	2	3	0
6	45	N/A	5	0	0	1	0
4.5	48	N/A	1	0	0	1	0
4.5	45	N/A	1	0	0	1	0
5	47	Yes	1	0	0	1	0
6	53	No	4	0	0	1	0

5.63	57	N/A	1	0	0	1	0
5.5	49	Yes	1	0	0	1	0
5.63	49	Yes	6	0	1	1	0
5.63	58	N/A	3	1	0	0	0
5	49	Yes	1	0	0	1	0
5.5	44	Yes	1	0	0	1	0
5.63	46	No	1	1	0	0	0
5.14	45	N/A	1	0	0	1	0
4.5	49	Yes	6	0	1	1	0
6	58	N/A	2	1	0	0	0
5.14	46	N/A	1	0	0	0	0
5	46	Yes	1	0	0	1	0
6	56	N/A	4	0	0	1	0
5.14	45	Yes	5	0	0	1	0
5.14	57	N/A	1	0	0	1	0
6	46	N/A	1	0	0	1	0
5.5	42	No	1	0	0	1	0
6	47	No	2	1	0	0	0
6	42	N/A	4	0	0	1	0
4.5	NA	N/A	3	1	0	0	0
6	45	Yes	2	1	0	0	0
4.5	49	N/A	2	1	0	0	0
5.14	52	N/A	3	1	0	0	0
4	55	No	2	0	0	0	0

GH5	GH8	GH9	GH10	GH12	GH13	GH15	GH16	GH20
2	0	0	0	0	0	0	2	0
0	0	0	0	0	0	0	0	0
1	0	0	0	0	0	0	0	0
0	0	0	0	0	0	0	0	0
0	0	0	0	0	0	0	0	0
3	0	0	0	0	0	0	0	0
0	0	0	0	0	0	0	1	1
0	0	0	1	0	1	0	0	0
0	0	0	0	0	0	0	0	0
0	0	0	1	0	0	0	0	0
0	0	0	0	0	0	0	0	0
0	0	0	0	0	0	0	0	0
2	0	0	0	0	0	0	2	1
0	0	0	0	0	0	0	0	0
1	0	0	0	0	0	0	0	0
0	0	0	0	0	0	0	0	0
1	0	0	0	0	0	0	0	0
1	0	0	0	0	0	0	0	0
0	0	0	1	0	0	0	0	0
0	0	0	0	0	0	0	0	0
0	0	0	0	0	0	0	0	0
0	0	0	1	0	0	0	0	0
0	0	0	0	0	0	0	0	0
0	0	0	0	1	0	0	0	0
0	0	0	0	1	0	0	0	0
0	0	0	0	0	1	0	1	0
0	0	0	1	0	0	0	0	0
0	0	0	0	0	0	0	0	0
0	0	0	0	0	0	0	0	0
0	0	0	1	0	0	0	0	0
1	0	0	0	0	0	0	0	0
1	0	0	0	0	0	0	0	0
1	0	0	0	0	0	0	0	0
0	0	1	0	0	0	0	0	0
0	0	1	0	0	0	0	0	0
1	0	0	0	0	0	0	2	0
1	0	0	0	0	0	0	0	0
0	0	0	0	0	1	0	0	0
0	0	0	0	0	1	0	0	0
0	1	0	0	0	0	0	0	0
1	0	0	0	0	0	0	1	0
0	0	0	0	0	0	0	0	0
0	0	0	0	0	0	0	0	0
0	0	0	0	0	0	0	0	0
1	1	0	0	0	0	0	0	0
1	1	0	0	0	0	0	0	0

1	0	0	0	0	0	0	2	0
0	0	0	0	0	0	0	1	0
0	0	0	0	0	0	0	1	0
0	0	0	0	0	0	0	0	0
0	0	1	0	0	0	0	0	0
1	0	0	0	0	0	0	1	0
1	0	0	0	0	0	0	0	0
0	0	1	0	0	0	0	0	0
0	0	0	2	0	0	0	0	0
0	0	0	0	0	1	0	0	0
0	0	0	0	0	1	0	1	0
0	0	0	0	0	1	0	0	0
1	0	0	0	0	0	0	0	0
1	0	0	0	0	0	0	0	0
0	0	0	1	0	0	0	0	0
0	0	0	0	0	0	0	1	0
0	0	0	2	0	0	0	0	0
0	0	0	0	0	0	0	3	0
0	0	0	0	0	0	0	1	0
0	0	0	3	0	0	0	0	0
0	0	0	2	0	0	0	0	0
0	0	0	0	0	1	0	0	0
1	1	0	0	0	0	0	0	0
0	0	0	0	0	0	0	1	0
0	0	0	0	0	0	0	0	0
0	0	0	0	0	0	0	0	0
0	0	0	0	0	1	0	0	0
2	0	0	0	0	0	0	0	0
1	0	0	0	0	0	0	0	0
0	0	0	0	0	0	0	0	0
1	0	0	0	0	0	0	0	0
0	0	0	2	0	0	0	0	0
1	0	0	0	0	1	0	0	0
1	0	0	0	0	0	0	2	0
0	0	0	1	0	0	0	0	0
0	0	0	0	0	0	0	0	0
0	0	0	1	0	0	0	0	0
1	0	0	0	0	0	0	0	0
1	2	0	0	0	0	0	0	0
1	0	0	0	0	0	0	0	0
1	1	0	0	0	0	0	0	0
0	0	0	0	0	0	0	0	0
0	0	0	0	0	0	0	0	0
0	0	0	0	0	0	0	0	0
0	0	0	3	0	0	0	0	0
0	0	0	1	0	0	0	0	0
0	0	0	0	0	0	0	0	0

0	0	0	1	0	0	0	0	0
0	0	0	0	0	0	0	0	0
0	0	0	0	0	0	0	0	0
0	0	0	0	0	1	0	0	0
0	0	0	0	0	0	0	0	0
0	0	0	0	0	0	0	0	0
0	0	0	0	0	0	0	0	0
0	0	0	0	0	0	0	0	0
0	0	0	0	0	0	0	0	0
0	0	0	0	0	1	1	0	0
0	0	0	0	0	0	0	0	0
1	0	0	0	0	0	0	1	1
1	0	0	0	0	0	0	0	0
0	0	0	0	0	0	0	0	0
0	0	0	0	0	0	0	1	0
1	0	0	0	0	0	0	0	0
0	0	0	0	0	0	0	0	0
0	0	0	0	0	0	0	0	0
0	0	0	0	0	0	0	0	0
0	0	0	0	0	0	0	0	0
0	0	0	0	0	0	0	1	0
0	0	0	0	0	0	0	0	0
1	0	0	0	0	0	0	0	0
1	0	0	0	0	0	0	0	0
0	0	0	0	0	0	1	0	0
2	0	0	0	0	0	0	0	0
0	0	0	0	0	0	0	0	0
0	0	0	0	0	0	0	0	0
0	0	0	0	0	0	0	1	1
0	0	0	0	0	0	0	0	0
0	0	0	0	0	0	0	0	0
0	0	0	1	0	0	0	0	0
0	0	0	1	0	0	0	0	0
0	0	0	0	0	0	0	0	0
0	0	0	0	0	0	0	1	1
0	0	0	0	0	0	0	0	0
1	0	0	0	0	0	0	1	0
1	0	0	0	0	0	0	1	0
0	0	0	1	0	0	0	0	0
0	0	0	0	0	0	0	0	0
0	0	0	0	0	0	0	0	0
1	0	1	0	0	0	0	0	0
0	0	0	0	0	0	0	0	0
0	0	0	0	0	0	0	0	0
0	0	0	0	0	0	0	0	0
0	0	0	0	0	0	0	0	0
0	0	0	0	0	0	0	1	0

0	0	0	0	0	0	0	0	0
0	0	0	0	0	0	0	0	0
1	0	0	1	0	0	0	0	0
0	0	0	0	0	1	0	0	0
0	0	0	0	0	0	0	0	0
0	0	0	0	0	0	0	0	0
0	0	0	0	0	0	0	0	0
0	0	0	0	0	0	0	0	0
1	0	0	1	0	0	0	0	0
0	0	0	0	0	1	0	0	0
1	0	0	0	0	0	0	0	0
0	0	0	0	0	0	0	0	0
0	0	0	0	0	0	0	1	0
0	0	0	0	0	0	0	1	0
0	0	0	0	0	0	0	0	0
0	0	0	0	0	0	0	0	0
0	0	0	0	0	0	0	0	0
0	0	0	0	0	1	0	0	0
0	0	0	0	0	0	0	1	0
0	0	0	0	0	0	0	0	0
0	0	0	0	0	1	0	0	0
0	0	0	0	0	0	0	0	0
0	0	0	0	0	1	0	0	0
2	0	0	0	0	0	0	0	0

GH23	GH26	GH27	GH30	GH31	GH35	GH36	GH38	GH39
0	0	0	1	0	0	0	0	0
0	0	0	0	0	0	0	0	0
0	0	0	0	0	0	0	0	0
0	0	0	0	0	0	0	0	0
0	0	0	0	0	0	0	0	0
0	0	0	0	0	0	0	0	0
0	0	0	0	0	0	0	0	0
0	0	0	0	0	0	0	0	0
0	0	1	0	0	0	0	0	0
0	0	0	0	0	0	0	0	0
0	0	0	0	0	0	0	0	0
0	0	0	0	0	0	0	0	0
0	0	0	0	0	0	0	0	0
0	0	0	0	0	0	0	0	0
0	0	0	0	0	0	0	0	0
0	0	0	0	0	0	0	0	0
0	0	0	0	0	0	0	0	0
1	0	0	0	0	0	0	0	0
0	0	0	0	0	0	0	0	0
0	0	0	0	0	0	0	0	0
0	0	0	0	0	0	0	0	0
0	0	0	0	0	0	0	0	0
0	0	0	0	0	0	0	0	0
0	0	0	0	0	0	0	0	0
0	0	0	0	0	0	0	0	0
0	0	0	0	0	0	0	0	0
0	0	0	0	0	0	0	0	0
0	0	0	0	0	0	0	0	0
0	0	0	0	0	0	0	0	0
0	0	0	1	0	0	0	0	0
1	0	1	0	0	0	0	0	0
1	0	1	0	0	0	0	0	0
0	0	1	0	0	0	0	0	0
0	0	0	0	0	0	0	0	0
0	0	0	0	0	0	0	0	0
0	1	0	0	0	0	0	0	0
0	0	0	0	0	0	0	0	0
1	0	0	0	0	0	0	0	0
1	0	0	0	0	0	0	0	0
0	0	0	0	0	0	0	0	0
0	0	0	0	0	0	0	0	0
0	0	0	0	0	0	0	0	0
0	0	0	0	1	0	0	0	0
0	0	0	0	0	0	0	0	0
0	0	1	0	0	0	0	0	0
0	0	1	0	0	0	0	0	0

0	1	0	0	0	0	0	0	0
0	0	0	0	0	0	0	0	0
0	0	0	0	0	0	0	0	0
0	0	0	0	0	0	0	0	0
0	0	0	0	0	0	0	0	0
0	0	0	0	0	0	0	0	0
1	0	1	0	0	0	0	0	0
0	0	0	0	0	0	0	0	0
0	0	0	2	0	0	0	0	1
0	0	0	0	0	0	0	0	0
0	0	0	0	0	0	0	0	0
0	0	0	0	0	0	0	0	0
1	0	1	0	0	0	0	0	0
0	0	1	0	0	0	0	0	0
0	0	0	2	0	0	0	0	1
0	0	0	0	0	0	0	0	0
0	0	0	2	0	0	0	0	1
0	0	0	0	0	0	0	0	0
1	0	0	0	0	0	0	0	0
0	0	0	0	2	0	0	0	0
0	0	0	2	0	0	0	0	1
0	0	0	0	0	0	0	0	0
0	0	1	0	0	0	0	0	0
0	0	0	0	0	0	0	0	0
0	0	0	0	0	0	0	0	0
0	0	0	0	0	0	0	0	0
0	0	0	0	0	0	0	0	0
0	0	0	1	0	0	0	0	0
0	0	1	0	0	0	0	0	0
0	0	0	0	0	0	0	0	0
0	0	0	0	0	0	0	0	0
0	0	0	0	0	0	0	0	0
0	0	0	0	0	0	0	0	0
0	0	0	0	0	0	0	0	0
0	0	0	2	0	0	0	0	1
0	0	0	0	0	0	0	0	0
0	0	0	2	0	0	0	0	1
0	0	1	0	0	0	0	0	0
0	0	1	0	0	0	0	0	0
1	0	1	0	0	0	0	0	0
0	0	1	0	0	0	0	0	0
0	0	0	0	0	0	0	0	0
0	0	0	0	0	0	0	0	0
0	0	0	0	1	0	0	0	0
0	0	0	0	1	0	0	0	0
0	0	0	2	0	0	0	0	1
0	0	0	0	0	0	0	0	0

0	0	0	1	0	0	0	0	0
0	0	0	0	1	0	0	0	0
0	0	0	0	0	0	0	0	0
0	0	0	0	0	0	0	0	0
0	0	0	0	0	0	0	0	0
0	0	0	0	0	0	0	0	0
0	0	0	0	0	0	0	0	0
0	0	0	0	0	0	0	0	1
0	0	0	0	0	0	0	0	0
0	0	0	0	1	0	0	0	0
0	0	0	0	0	0	0	0	0
0	0	0	0	0	0	0	0	0
0	0	0	0	0	0	0	0	0
0	0	0	0	0	0	0	0	0
0	0	0	0	0	0	0	0	0
0	0	0	0	0	0	0	0	0
0	0	0	0	0	0	0	0	0
0	0	0	0	0	0	0	0	0
0	0	0	0	0	0	0	0	0
0	0	0	0	0	0	0	0	0
0	0	0	0	0	0	0	0	0
0	0	0	0	0	0	0	0	0
0	0	0	0	0	0	0	0	1
0	0	0	0	1	0	0	0	0
0	0	0	0	1	0	0	0	0
0	0	0	0	0	0	0	0	0
0	0	0	0	0	0	0	0	0
0	0	0	0	0	0	0	0	0
0	0	0	0	0	0	0	0	0
0	0	0	1	0	0	0	0	0
0	0	0	0	0	0	0	0	0
0	0	0	0	0	0	0	0	0
0	0	0	0	0	0	0	0	0
0	0	0	0	0	0	0	0	0
0	0	0	0	0	0	0	0	0
0	0	0	0	0	0	0	0	0
0	0	0	1	0	0	0	0	0
0	0	0	1	0	0	0	0	0
0	0	0	0	0	0	0	0	0
0	0	0	0	0	0	0	0	0
0	0	0	0	0	0	0	0	0
0	0	0	0	1	0	0	0	0
0	0	0	0	0	0	0	0	0
0	0	0	0	0	0	0	0	0
0	0	0	0	0	0	0	0	0
0	0	0	0	0	0	0	0	0
0	0	0	1	0	0	0	0	0

0	0	0	0	0	0	0	0	0
0	0	0	0	0	0	0	0	0
0	0	0	0	0	0	0	0	0
0	0	0	0	0	0	0	0	0
0	0	0	0	0	0	0	0	0
0	0	0	0	0	0	0	0	0
0	0	0	0	0	0	0	0	0
0	0	0	0	0	0	0	0	0
0	0	0	0	0	0	0	0	0
0	0	0	0	0	0	0	0	0
0	0	0	0	0	0	0	0	0
0	0	0	0	0	0	0	0	0
0	0	0	1	0	0	0	0	0
0	0	0	1	0	1	0	0	0
0	0	0	0	0	0	0	0	0
0	0	0	0	0	0	0	0	0
0	0	0	0	0	0	0	0	0
0	0	0	0	0	0	0	0	0
0	0	0	1	0	0	0	0	0
0	0	0	0	0	0	1	1	0
0	0	0	0	0	0	0	0	0
0	0	0	0	0	0	0	0	0
1	0	0	0	0	0	0	0	0
0	0	0	0	0	0	0	0	0

GH43	GH44	GH51	GH53	GH55	GH63	GH65	GH66	GH67
0	0	0	0	0	0	0	0	0
0	0	0	0	0	0	0	0	0
0	0	0	0	0	0	0	0	0
0	0	0	0	0	0	0	0	0
0	0	0	0	0	0	0	0	0
0	0	0	0	0	0	0	0	0
0	0	0	0	1	0	0	0	0
0	0	0	0	0	0	0	0	0
0	0	0	0	0	0	0	0	0
0	0	0	0	0	0	0	0	0
0	0	0	0	0	0	0	0	0
0	0	0	0	0	0	0	0	0
0	0	0	0	0	0	0	0	0
0	0	0	0	0	0	0	0	0
0	0	0	0	0	0	0	0	0
0	0	0	0	0	0	0	0	0
0	0	0	0	0	0	0	0	0
0	0	0	0	0	0	0	0	0
3	0	0	0	0	0	0	0	0
1	0	0	0	0	0	0	0	0
0	0	0	0	0	0	0	0	0
0	0	1	0	0	0	0	0	0
0	0	0	0	0	0	0	0	0
0	0	0	0	0	0	0	0	0
0	0	0	0	0	0	0	0	0
0	0	0	0	0	0	0	0	0
0	0	0	0	0	0	0	0	0
0	0	0	0	0	0	0	0	0
0	0	0	1	0	1	0	0	0
0	0	0	0	0	0	0	0	0
0	1	0	0	0	0	0	0	0
0	0	0	0	0	0	0	0	0
1	0	0	0	0	0	0	0	0
0	0	0	0	0	0	0	0	0
0	0	0	0	0	0	0	0	0
0	0	0	0	0	0	0	0	0
1	0	0	0	0	0	0	0	0
0	0	0	0	0	0	0	0	0
0	0	0	0	0	0	0	0	0
0	0	0	0	0	0	0	0	0
0	0	0	0	0	0	0	0	0
0	0	0	0	0	0	0	0	0
0	0	0	0	0	0	0	0	0
0	0	0	0	0	0	0	0	0
1	0	0	0	0	0	0	0	0
1	0	0	0	0	0	0	0	0

0	0	0	0	0	0	0	0	0
0	0	0	0	0	0	0	0	0
0	0	0	0	0	0	0	0	0
0	0	0	0	0	0	0	0	0
0	0	0	0	0	0	0	0	0
0	0	0	0	0	0	0	0	0
1	0	0	0	0	0	0	0	0
0	0	0	0	0	0	0	0	0
1	0	0	0	0	0	1	0	0
0	0	0	0	0	0	0	0	0
0	0	0	0	0	0	0	0	0
0	0	0	0	0	0	0	0	0
0	1	0	0	0	0	0	0	0
1	0	0	0	0	0	0	0	0
0	0	0	0	0	0	0	0	0
0	0	0	0	0	0	0	0	0
1	0	0	0	0	0	1	0	0
0	0	0	0	0	0	0	0	0
0	0	0	0	0	0	0	0	0
3	0	0	0	0	0	0	0	0
1	0	0	0	0	0	1	0	0
0	0	0	0	0	0	0	0	0
1	0	0	0	0	0	0	0	0
0	0	0	0	0	0	0	0	0
0	0	0	0	0	0	0	0	0
0	0	0	0	0	0	0	0	0
0	0	0	0	0	0	0	0	0
0	0	0	0	0	0	0	0	0
0	0	0	0	0	0	0	0	0
0	0	0	0	0	0	0	0	0
1	0	0	0	0	0	0	0	0
2	0	0	0	0	0	0	0	1
0	0	0	0	0	0	0	0	0
0	0	0	0	0	0	0	0	0
0	0	0	0	0	0	0	0	0
0	0	0	0	0	0	0	0	0
0	0	0	0	0	0	0	0	0
0	0	0	0	0	0	0	0	0
1	0	0	0	0	0	0	0	0
0	0	0	0	0	0	0	0	0
1	0	0	0	0	0	0	0	0
0	0	0	0	0	0	0	0	0
0	0	0	0	0	0	0	0	0
0	0	0	0	0	0	0	0	0
3	0	0	0	0	0	0	0	1
0	0	0	0	0	0	0	0	0
0	0	0	0	0	0	0	0	0

0	0	0	0	0	0	0	0	0
0	0	0	0	0	0	0	0	0
0	0	0	0	0	0	0	0	0
0	0	0	0	0	0	0	1	0
0	0	0	0	0	0	0	0	0
0	0	0	0	0	0	0	0	0
0	0	0	0	0	0	0	0	0
0	0	0	0	0	0	0	0	0
0	0	0	0	0	0	0	0	0
0	0	0	0	0	0	0	0	0
0	0	0	0	0	0	0	0	0
0	0	0	0	0	0	0	0	0
0	0	0	0	0	0	0	0	0
0	0	0	0	0	0	0	0	0
0	0	0	0	0	0	0	0	0
0	0	0	0	0	0	0	0	0
0	0	0	0	0	0	0	0	0
0	0	0	0	0	0	0	0	0
0	0	0	0	0	0	0	0	0
0	0	0	0	0	0	0	0	0
0	0	0	0	1	0	0	0	0
0	0	0	0	0	0	0	0	0
0	0	0	0	0	0	0	0	0
0	0	0	0	0	0	0	0	0
0	0	0	0	0	0	0	0	0
0	0	0	0	0	0	0	0	0
0	0	0	0	0	0	0	0	0
0	0	0	0	0	0	0	0	0
0	0	0	0	1	0	0	0	0
0	0	0	1	0	0	0	0	0
0	0	0	0	0	0	0	0	0
0	0	0	0	0	0	0	0	0
0	0	0	0	0	0	0	0	0
0	0	0	0	0	0	0	0	1
0	0	0	0	0	0	0	0	0
0	0	0	0	0	0	0	0	0
0	0	0	0	0	0	0	0	0
0	0	0	0	0	0	0	0	0
1	0	0	0	0	0	0	0	1
0	0	0	0	0	0	0	0	0
0	0	0	0	0	0	0	0	0
0	0	0	0	0	0	0	0	0
1	0	0	0	0	0	0	0	0
0	0	0	0	0	0	0	0	0
0	0	0	0	0	0	0	0	0
0	0	0	0	0	0	0	0	0
0	0	0	0	0	0	0	0	0

0	0	0	0	0	0	0	0	0
0	0	0	0	0	0	0	0	0
0	0	0	0	0	0	1	0	1
0	0	0	0	0	0	1	0	0
0	0	0	0	0	0	0	0	0
0	0	0	0	0	0	0	0	0
0	0	0	0	0	0	0	0	0
0	0	0	0	0	0	0	0	0
0	0	0	0	0	0	1	0	1
0	0	0	0	0	0	0	0	0
0	0	0	0	0	0	0	0	0
0	0	0	0	0	0	0	0	0
0	0	0	0	0	0	0	0	0
0	0	0	0	0	0	0	0	0
0	0	0	0	0	0	0	0	0
0	0	0	0	0	0	0	0	0
0	0	0	0	0	0	0	0	0
0	0	0	0	0	0	0	0	0
0	0	0	0	0	0	0	0	0
0	0	0	0	0	0	0	0	0
0	0	0	0	0	0	0	0	0
0	0	0	1	0	0	0	0	0
0	0	0	0	0	0	0	0	0
0	0	0	0	0	0	0	0	0

GH73	GH78	GH87	GH93	GH94	GH95	GH97	GH105	GH109
0	0	0	0	0	0	0	0	0
0	0	0	0	0	0	0	0	0
0	0	0	0	0	0	0	0	0
0	0	0	0	0	0	0	0	0
0	0	0	0	0	0	0	0	0
0	0	0	0	0	0	0	0	0
0	0	0	0	0	0	0	0	0
0	0	0	0	0	0	0	0	0
0	0	0	0	0	0	0	0	0
0	0	0	0	0	0	0	0	0
0	0	0	0	0	0	0	0	0
0	0	0	0	0	0	0	0	0
0	0	0	0	0	0	0	0	0
0	0	0	0	1	0	0	0	0
0	0	0	0	0	0	0	0	0
0	0	0	0	0	0	0	0	0
0	0	0	0	0	0	0	0	0
0	0	0	0	0	0	0	0	0
0	0	0	0	0	0	0	0	0
0	0	0	0	0	0	0	0	0
0	0	0	0	0	0	0	0	0
0	0	0	0	0	0	0	0	0
0	0	0	0	0	0	0	0	0
0	0	0	0	0	0	0	0	0
0	0	0	0	0	0	0	0	0
0	0	0	0	0	0	0	0	0
0	0	0	0	0	0	0	0	0
0	0	0	0	0	0	0	0	0
0	1	0	0	0	0	0	0	0
0	0	0	0	0	0	0	0	0
0	0	0	0	0	0	0	0	0
0	0	0	0	0	0	0	0	0
0	0	0	0	0	0	0	0	0
0	0	0	0	0	0	0	0	0
0	0	0	0	0	0	0	0	0
0	0	0	0	0	0	0	0	0
0	0	0	0	0	0	0	0	0
0	0	0	0	0	0	0	0	0
0	0	0	0	0	0	0	0	0
0	0	0	0	0	0	0	0	0
0	0	0	0	0	0	0	0	0
0	0	0	0	0	0	0	0	0
0	0	0	0	0	0	0	0	0
0	0	0	0	0	0	0	0	0
0	0	0	0	0	0	0	0	0
0	0	0	0	0	0	0	0	0

0	0	0	0	0	0	0	0	0
0	1	0	0	0	0	0	0	0
0	0	0	0	0	0	0	0	0
0	0	0	0	0	0	0	0	0
0	0	0	0	0	0	0	0	0
0	0	0	0	0	0	0	0	0
0	0	0	0	0	0	0	0	0
0	0	0	0	0	0	0	0	0
0	0	0	0	0	0	0	0	0
0	0	0	0	0	0	0	0	0
0	0	0	0	0	0	0	0	0
0	0	0	0	0	0	0	0	0
0	0	0	0	0	0	0	0	0
0	0	0	0	0	0	0	0	0
0	0	0	0	0	0	0	0	0
0	0	0	0	0	0	0	0	0
0	0	0	0	0	0	0	0	0
0	0	0	0	0	0	0	0	0
0	0	0	0	0	0	0	0	0
0	0	0	0	0	1	1	0	0
0	0	0	0	0	0	0	0	0
0	0	0	0	0	0	0	0	0
0	0	0	0	0	0	0	0	0
0	0	0	0	0	0	0	0	0
0	0	0	0	0	0	0	0	0
0	1	0	0	0	0	0	0	0
0	0	0	0	0	0	0	0	0
0	0	0	0	0	0	0	0	0
0	0	0	0	0	0	0	0	0
0	0	0	0	0	0	0	0	0
0	0	0	0	0	0	0	0	0
0	0	0	0	0	0	0	0	0
0	0	0	0	1	0	0	1	0
0	0	0	0	0	0	0	0	0
0	0	0	0	0	0	0	0	0
0	0	0	0	0	0	0	0	0
0	0	0	0	0	0	0	0	0
0	0	0	0	0	0	0	0	0
0	0	0	0	0	0	0	0	0
0	0	0	0	0	0	0	0	0
0	0	0	0	0	0	0	0	0
0	0	0	0	0	0	0	0	0
0	1	0	0	0	0	0	0	0
0	0	0	0	0	0	0	0	0
0	0	0	0	0	0	1	0	0
0	0	0	0	0	0	0	0	0
0	1	0	0	0	0	0	0	0

0	0	0	0	0	0	0	0	0
0	0	0	0	0	0	0	0	0
0	0	0	0	0	0	0	0	0
0	0	0	0	0	0	0	0	0
0	0	0	0	0	0	0	0	0
0	0	0	0	0	0	0	0	0
0	0	0	0	0	0	0	0	0
0	0	0	0	0	0	0	0	0
0	0	0	0	0	0	0	0	0
0	0	0	0	0	0	0	0	0
0	0	0	0	0	0	0	0	0
0	0	0	0	0	0	0	0	0
0	0	0	0	0	0	0	0	0
0	0	0	0	1	0	0	0	0
0	0	0	0	0	0	0	0	0
0	0	0	0	0	0	0	0	0
0	0	0	0	0	0	0	0	0
0	0	0	0	0	0	0	0	0
0	0	0	1	0	0	0	0	0
0	0	0	0	0	0	0	0	0
0	0	0	0	0	0	0	0	0
0	0	0	0	0	0	0	0	0
0	0	0	0	0	0	0	0	0
0	0	0	0	0	0	0	0	0
0	0	0	0	0	0	0	0	1
0	0	0	0	0	0	0	0	0
0	1	0	0	0	0	0	0	0
0	0	0	0	0	0	0	0	0
0	0	0	0	0	0	0	0	0
0	0	0	1	0	0	0	0	0
0	0	0	0	0	0	0	0	0
0	0	0	0	0	0	0	0	0
1	0	1	0	0	0	0	0	0
0	0	0	0	0	0	0	0	0
0	0	0	0	0	0	0	0	0
0	0	0	0	0	0	0	0	0
0	0	0	0	0	0	0	0	0
0	0	0	0	0	0	0	0	0
0	0	0	0	0	0	0	0	0
0	0	0	0	0	0	0	0	0
0	0	0	0	0	0	0	0	0
0	0	0	0	0	1	0	0	0
0	1	0	0	0	0	0	0	0
0	0	0	0	0	0	0	0	0
0	0	0	0	0	0	0	0	0
0	0	0	0	0	0	0	0	0
0	0	0	0	0	0	0	0	0

0	0	0	0	0	0	0	0	0
0	0	0	0	0	0	0	0	0
0	0	0	0	0	0	0	0	0
0	0	0	0	0	0	0	0	0
0	0	0	0	0	0	0	0	0
0	0	0	0	0	0	0	0	0
0	0	0	0	0	0	0	0	0
0	0	0	0	0	0	0	0	0
0	0	0	0	0	0	0	0	0
0	0	0	0	0	0	0	0	0
0	0	0	0	0	0	0	0	0
0	0	0	0	0	0	0	0	0
0	0	0	0	0	0	0	0	0
0	0	0	0	0	0	0	0	0
0	0	0	0	0	0	0	0	0
0	0	0	0	0	0	0	0	0
0	0	0	0	0	0	0	0	0
0	0	0	0	0	0	0	0	0
0	0	0	0	0	0	0	0	0
0	0	0	0	0	0	0	0	0
0	0	0	0	0	0	0	0	0
0	0	0	0	0	0	0	0	0
0	0	0	0	0	0	0	0	0
0	0	0	0	0	0	0	0	0

GH115	GH116	GH127	GH128	GH130	GH135	GH141	GH144	GH146
0	0	0	0	0	0	0	0	0
0	0	0	0	1	0	0	0	0
0	0	0	0	0	0	0	0	0
0	0	0	0	0	0	0	0	0
0	0	0	0	0	0	0	0	0
0	0	0	0	1	0	0	0	0
0	0	0	0	0	0	0	0	0
0	0	0	0	0	0	0	0	0
0	0	0	0	0	0	0	0	0
0	0	0	0	0	0	0	0	0
0	0	0	0	0	0	0	0	0
0	0	0	0	0	0	0	0	0
0	0	0	0	0	0	0	0	0
0	0	0	0	0	0	0	0	0
0	0	0	0	0	0	0	0	0
0	0	0	0	0	0	0	0	0
0	0	0	0	0	0	0	0	0
0	0	0	0	0	0	0	0	0
1	0	0	0	0	0	0	0	0
0	0	0	0	0	0	0	0	0
0	0	0	0	0	0	0	1	0
0	0	0	1	0	0	0	0	0
0	0	0	0	0	0	0	1	0
0	0	0	0	0	0	0	1	0
0	0	0	0	0	0	0	1	0
0	0	0	0	0	0	0	0	0
0	0	0	0	0	0	0	0	0
0	0	0	0	0	0	0	0	0
0	0	0	0	0	0	0	0	0
0	0	0	0	0	0	0	0	0
0	0	0	0	0	0	0	0	0
0	0	0	0	0	0	0	0	0
0	0	0	0	0	0	1	0	0
0	0	0	0	0	0	0	0	0
0	0	0	0	0	0	0	0	0
0	0	0	0	0	0	0	0	0
0	0	0	0	0	0	0	0	0
0	0	0	0	0	0	0	0	0
0	0	0	0	0	0	0	0	0
0	0	0	0	0	0	0	0	0
0	0	0	0	0	0	0	0	0
0	0	0	0	0	0	0	0	0
0	0	0	0	0	0	0	0	0
0	0	0	0	0	0	0	0	0
0	0	0	0	0	0	1	0	0
0	0	0	0	0	0	1	0	0

0	0	0	0	0	0	0	0	0
0	0	0	0	0	0	0	0	0
0	0	0	0	0	0	0	0	0
0	0	0	0	0	0	0	0	0
0	0	0	0	0	0	0	0	0
0	0	0	0	0	0	0	0	0
0	0	0	0	0	0	0	0	0
0	0	0	0	0	0	0	0	0
0	0	0	0	0	0	0	0	0
0	0	0	0	0	0	0	0	0
0	0	0	0	0	0	0	0	0
0	0	0	0	0	0	0	0	0
0	0	0	0	0	0	0	0	0
0	0	0	0	0	0	1	0	0
0	0	0	0	0	0	0	0	0
0	0	0	0	0	0	0	0	0
0	0	0	0	0	0	0	0	0
0	0	0	0	0	0	0	0	0
0	0	0	0	0	0	0	0	0
0	0	0	0	0	0	0	0	1
0	0	0	0	0	0	0	0	0
0	0	0	0	0	0	0	0	0
0	0	0	0	0	0	1	0	0
0	0	0	0	0	0	0	0	0
0	0	0	0	0	0	0	0	0
0	0	0	0	0	0	0	0	0
0	0	0	0	0	0	0	0	0
0	0	0	0	0	0	0	0	0
0	0	0	0	0	0	0	0	0
0	0	0	0	0	0	0	0	0
0	0	0	0	0	0	0	0	0
0	0	0	0	0	0	0	0	0
0	0	0	0	0	0	0	0	0
0	0	0	0	0	0	0	0	0
0	0	0	0	0	0	0	0	0
0	0	0	0	0	0	0	0	0
0	0	0	0	0	0	0	0	0
0	0	0	0	0	0	0	0	0
0	0	0	0	0	0	1	0	0
0	0	0	0	0	0	0	0	0
0	0	0	0	0	0	1	0	0
0	0	0	0	0	0	0	0	0
0	1	0	0	0	0	0	0	0
0	0	0	0	0	0	0	0	0
0	0	0	0	0	0	0	0	1
0	0	0	0	0	0	0	0	0
0	1	0	0	0	0	0	0	0

0	0	0	0	0	0	0	0	0
0	0	0	0	0	0	0	0	0
0	0	0	0	0	0	0	0	0
0	0	0	0	0	0	0	0	0
0	0	0	0	0	0	0	0	0
0	0	0	0	0	0	0	0	0
0	0	0	0	0	0	0	0	0
0	0	0	0	0	0	0	0	0
0	0	0	0	0	0	0	0	0
0	0	0	0	0	0	0	0	0
0	0	0	0	0	0	0	0	1
0	0	0	0	0	0	0	0	0
0	0	0	0	0	0	0	0	0
0	0	0	0	0	0	0	0	0
0	0	0	0	0	0	0	0	0
0	0	0	0	0	0	0	0	0
0	0	0	0	0	0	0	0	0
0	1	0	0	0	0	0	0	0
0	0	0	0	1	0	0	0	0
0	0	0	0	0	0	0	0	0
0	0	1	0	0	0	0	0	1
0	0	0	0	0	0	0	0	0
0	0	0	0	0	0	0	0	0
0	0	0	0	0	0	0	0	0
0	0	0	0	0	0	0	1	0
0	0	0	0	0	0	0	0	0
0	0	0	0	0	0	0	0	0
0	0	0	0	0	1	0	0	0
0	0	0	0	0	0	0	0	0
0	0	0	0	0	0	0	0	0
0	1	0	0	0	0	0	0	0
0	0	0	0	0	0	0	0	0
0	0	0	0	0	0	0	0	0
0	0	0	0	0	0	0	0	0
0	0	0	0	0	0	0	0	0
0	0	0	0	0	0	0	0	0
0	0	0	0	0	0	0	0	0
0	0	0	0	0	0	0	0	0
1	0	0	0	0	0	0	0	0
0	0	0	0	0	0	0	0	0
0	0	0	0	0	0	0	0	0
0	0	0	0	0	0	0	0	0
0	0	0	0	0	0	0	2	0
0	0	0	0	0	0	0	0	0
0	0	0	0	0	0	0	0	0
0	0	0	0	0	0	0	0	0
0	0	0	0	0	0	0	0	0

0	0	0	0	0	0	0	0	0
0	0	0	0	0	0	0	0	0
0	0	0	0	0	0	0	0	0
0	0	0	0	0	0	0	0	0
0	0	0	0	0	0	0	0	0
0	0	0	0	0	0	0	0	0
0	0	0	0	0	0	0	0	0
0	0	0	0	0	0	0	0	0
0	0	0	0	0	0	0	0	0
0	0	0	0	0	0	0	0	0
0	0	0	0	0	0	0	0	0
0	0	0	0	0	0	0	0	0
0	0	0	0	0	0	0	0	0
0	0	0	0	0	0	0	0	0
0	0	0	0	0	0	0	0	0
0	0	0	0	0	0	0	0	0
0	0	0	0	0	0	0	0	0
0	0	0	0	0	0	0	0	0
0	0	0	0	0	0	0	0	0
0	0	0	0	0	0	0	0	0
0	0	0	0	0	0	0	0	0
0	0	0	0	0	0	0	0	0
0	0	0	0	0	0	0	0	0
0	0	0	0	0	0	0	0	0

GH149
1
0
0
0
0
0
0
0
0
0
0
0
0
0
0
0
0
0
0
0
0
0
0
0
0
0
0
0
0
0
0
0
0
0
0
0
0
0
0
0
0
0
0
0
0
0

0
0
0
0
0
0
0
0
0
0
0
0
0
0
0
0
0
0
0
0
0
0
0
0
0
0
0
0
0
0
0
0
0
0
0
0
0
0
0
0
0
0
0
0
0
0
0

0
0
0
0
0
0
0
0
0
0
0
0
0
0
0
0
0
0
0
0
0
0
0
0
0
0
0
0
0
0
0
0
0
0
0
0
0
0
0
0
0
0
0
0
0
0
1

0
0
0
0
0
0
0
0
0
0
0
0
1
1
0
0
0
0
1
0
0
0
0
0
