# Supplementary material for: High-Throughput Recovery and Characterization of Metagenome-Derived Glycoside Hydrolase-Containing Clones as a Resource for Biocatalyst Development
Source: mSystems. 2019 Jun 4;4(4):e00082-19. doi: 10.1128/mSystems.00082-19 (PMC6550366; doi:10.1128/mSystems.00082-19)
Supplement: TABLE S3 [file mSystems.00082-19-st003.pdf]

Fosmid	LCAStar_pval
TolDC_59_K14	0
TolDC_59_J06	0
TolDC_59_J01	0.002
TolDC_59_E21	0
TolDC_56_L15	0.055
TolDC_56_H11	0
TolDC_55_H19	0.009
TolDC_50_P08	0.041
TolDC_50_B06	0.001
TolDC_46_B16	0.001
TolDC_41_A17	0.295
TolDC_39_M03	0.153
TolDC_38_E11	0.003
TolDC_35_I03	0.001
TolDC_32_D22	0.024
TolDC_31_E21	0
TolDC_30_J10	0
TolDC_30_A19	0.005
TolDC_25_I24	0
TolDC_22_J01	0.001
TolDC_22_A01	0
TOLDC_20_J14	0
TolDC_15_G15	0.005
TOLDC_15_E19	0.015
TOLDC_15_D05	0
TOLDC_15_C08	0.009
TolDC_10_A11	0.025
TOLDC_08_I17	0.001
TOLDC_06_L02	0.002
SCR03_04_B15	0.56
SCR03_01_L21	0.002
PWCG7_49_G20	0.096
PWCG7_33_K24	0.273
PWCG7_19_J20	0.088
PWCG7_19_I21	0.088
NR003_36_K13	0.046
NR003_09_O07	0.004
NR003_03_D21	0
NO002_07_F01	0.088
NO002_04_P09	0
NO002_01_J07	0
NO001_13_N07	0.129
NO001_10_L12	0
NO001_08_N01	0
NO001_08_K19	0.076
NO001_07_A13	0.009

NO001_04_B04	0.009
NO001_03_P09	0.009
NO001_01_I19	0.001
NO001_01_G23	0
NB001_23_D20	0
NB001_14_K20	0.009
NB001_13_B14	0
NB001_12_A01	0
NB001_03_I24	0.003
NapDC_53_D04	0
NapDC_52_E10	0.025
NapDC_21_E17	0.019
NapDC_20_D21	0.001
NA004_04_B18	0.003
NA002_01_B04	0.002
NA001_16_B03	0.016
NA001_11_K24	0.012
NA001_07_F24	0.306
NA001_07_E13	0.016
NA001_02_B17	0
NA001_01_P12	0.001
FOS62_47_P19	0.403
FOS62_47_J09	0.009
FOS62_47_H05	0.306
Fos62_47_F04	0.001
FOS62_47_B05	0.027
FOS62_46_L17	0.002
FOS62_46_E02	0.295
FOS62_46_D05	0.07
Fos62_44_J10	0.032
FOS62_44_F23	0
FOS62_44_E09	0.007
FOS62_44_A15	0
FOS62_43_O18	0.025
FOS62_43_J23	0
Fos62_43_J20	0.041
FOS62_43_F03	0.016
FOS62_43_C07	0.002
FOS62_42_K13	0
FOS62_42_D11	0
FOS62_41_N11	0
FOS62_41_L01	0
FOS62_41_K19	0.009
FOS62_41_K10	0.016
FOS62_41_I01	0
FOS62_41_D24	0.009
FOS62_41_C11	0

FOS62_41_A23	0.015
FOS62_40_G22	0.014
FOS62_40_E07	0.003
FOS62_38_N16	0.005
FOS62_38_G18	0.282
FOS62_38_D22	0.273
FOS62_38_C16	0.022
FOS62_38_A06	0.125
FOS62_37_N12	0.003
FOS62_37_N04	0.014
FOS62_37_C18	0.065
FOS62_36_K01	0
FOS62_36_J17	0
FOS62_35_C14	0.009
FOS62_34_K14	0.065
FOS62_34_J06	0.009
FOS62_34_D13	0.244
FOS62_30_N01	0.002
FOS62_30_L24	0
FOS62_30_J11	0
FOS62_30_H03	0.002
FOS62_30_E20	0
FOS62_29_F15	0.009
FOS62_29_C04	0.055
FOS62_28_K23	0.009
FOS62_28_A14	0
FOS62_27_P24	0
FOS62_27_N22	0.046
FOS62_27_M17	0.035
FOS62_26_L14	0.005
FOS62_26_K16	0
FOS62_26_K06	0.03
FOS62_26_C24	0
FOS62_26_C23	0
FOS62_25_O06	0.003
FOS62_25_L08	0.009
FOS62_25_H06	0.001
FOS62_24_P09	0.003
FOS62_24_L18	0.003
FOS62_24_J23	0
FOS62_23_J07	0
FOS62_23_B24	0.027
FOS62_22_C08	0.016
FOS62_21_J05	0.009
Fos62_21_D16	0
FOS62_21_B24	0.038
FOS62_10_P15	0.104

FOS62_10_O15	0.104
FOS62_08_G04	0.035
FOS62_08_D12	0
FOS62_08_C22	0.035
CO182_36_O04	0.012
CO182_36_O01	0.024
CO182_24_J12	0.313
CO182_11_I14	0.008
CO004_10_P05	0.015
CO004_05_B17	0.024
CO003_01_D22	0.249
CO002_07_L07	0.001
CG23A_09_O05	0.006
CG23A_01_C20	0.006
CB006_08_D19	0
CB006_04_L11	0.003
CB005_08_O01	0
CB004_10_B20	0.001
CB004_07_C21	0.001
CB003_08_B11	0
CB002_04_H07	0.027
40500_12_L11	0.002
12500_09_F02	None
12200_16_F10	0.016

LCAStar_assgnment
root (1);cellular organisms (131567);Bacteria (2)
root (1);cellular organisms (131567);Bacteria (2)
root (1);cellular organisms (131567);Bacteria (2)
root (1);cellular organisms (131567);Bacteria (2)
root (1);cellular organisms (131567);Bacteria (2)
root (1);cellular organisms (131567);Bacteria (2)
root (1);cellular organisms (131567);Bacteria (2)
root (1);cellular organisms (131567);Bacteria (2)
root (1);cellular organisms (131567);Bacteria (2)
root (1);cellular organisms (131567);Bacteria (2)
root (1);cellular organisms (131567);Bacteria (2);FCB group (1783270);Bacteroidetes/Chlorobi group (68336);Bact
root (1);cellular organisms (131567);Bacteria (2);FCB group (1783270);Bacteroidetes/Chlorobi group (68336);Bact
root (1);cellular organisms (131567);Bacteria (2)
root (1);cellular organisms (131567);Bacteria (2)
root (1);cellular organisms (131567);Bacteria (2)
root (1);cellular organisms (131567);Bacteria (2)
root (1);cellular organisms (131567);Bacteria (2)
root (1);cellular organisms (131567);Bacteria (2)
root (1);cellular organisms (131567);Bacteria (2)
root (1);cellular organisms (131567);Bacteria (2)
root (1);cellular organisms (131567);Bacteria (2)
root (1);cellular organisms (131567);Bacteria (2)
root (1);cellular organisms (131567);Bacteria (2)
root (1);cellular organisms (131567);Bacteria (2)
root (1);cellular organisms (131567);Bacteria (2)
root (1);cellular organisms (131567);Bacteria (2)
root (1);cellular organisms (131567);Bacteria (2)
root (1);cellular organisms (131567);Bacteria (2)
root (1);cellular organisms (131567);Bacteria (2);FCB group (1783270);Bacteroidetes/Chlorobi group (68336);Bact
root (1);cellular organisms (131567);Bacteria (2)
root (1);cellular organisms (131567);Bacteria (2)
root (1);cellular organisms (131567);Bacteria (2)
root (1);cellular organisms (131567);Bacteria (2);FCB group (1783270);Bacteroidetes/Chlorobi group (68336);Bact
root (1);cellular organisms (131567);Bacteria (2);FCB group (1783270);Bacteroidetes/Chlorobi group (68336);Bact
root (1);cellular organisms (131567);Bacteria (2);FCB group (1783270);Bacteroidetes/Chlorobi group (68336);Bact
root (1);cellular organisms (131567);Bacteria (2)
root (1);cellular organisms (131567);Bacteria (2)
root (1);cellular organisms (131567);Bacteria (2)
root (1);cellular organisms (131567);Bacteria (2)
root (1);cellular organisms (131567);Bacteria (2)
root (1);cellular organisms (131567);Bacteria (2)
root (1);cellular organisms (131567);Bacteria (2);Terrabacteria group (1783272);Actinobacteria (201174)
root (1);cellular organisms (131567);Bacteria (2)
root (1);cellular organisms (131567);Bacteria (2)
root (1);cellular organisms (131567);Bacteria (2)
root (1);cellular organisms (131567);Bacteria (2)

root (1);cellular organisms (131567);Bacteria (2)
root (1);cellular organisms (131567);Bacteria (2)
root (1);cellular organisms (131567);Bacteria (2)
root (1);cellular organisms (131567);Bacteria (2)
root (1);cellular organisms (131567);Bacteria (2)
root (1);cellular organisms (131567);Bacteria (2);Terrabacteria group (1783272);Actinobacteria (201174)
root (1);cellular organisms (131567);Bacteria (2)
root (1);cellular organisms (131567);Bacteria (2)
root (1);cellular organisms (131567);Bacteria (2)
root (1);cellular organisms (131567);Bacteria (2)
root (1);cellular organisms (131567);Bacteria (2);FCB group (1783270);Bacteroidetes/Chlorobi group (68336);Bact
root (1);cellular organisms (131567);Bacteria (2)
root (1);cellular organisms (131567)
root (1);cellular organisms (131567);Bacteria (2)
root (1);cellular organisms (131567);Bacteria (2)
root (1);cellular organisms (131567);Bacteria (2)
root (1);cellular organisms (131567);Bacteria (2)
root (1);cellular organisms (131567);Bacteria (2)
root (1);cellular organisms (131567);Bacteria (2)
root (1);cellular organisms (131567);Bacteria (2)
root (1);cellular organisms (131567);Bacteria (2)
root (1);cellular organisms (131567);Bacteria (2);Terrabacteria group (1783272)
root (1);cellular organisms (131567);Bacteria (2)
root (1);cellular organisms (131567);Bacteria (2)
root (1);cellular organisms (131567);Bacteria (2)
root (1);cellular organisms (131567);Bacteria (2)
root (1);cellular organisms (131567);Bacteria (2)
root (1);cellular organisms (131567);Bacteria (2);FCB group (1783270);Bacteroidetes/Chlorobi group (68336);Bact
root (1);cellular organisms (131567);Bacteria (2);Terrabacteria group (1783272)
root (1);cellular organisms (131567);Bacteria (2)
root (1);cellular organisms (131567);Bacteria (2)
root (1);cellular organisms (131567);Bacteria (2)
root (1);cellular organisms (131567);Bacteria (2)
root (1);cellular organisms (131567);Bacteria (2)
root (1);cellular organisms (131567);Bacteria (2)
root (1);cellular organisms (131567);Bacteria (2)
root (1);cellular organisms (131567);Bacteria (2)
root (1);cellular organisms (131567);Bacteria (2)
root (1);cellular organisms (131567);Bacteria (2)
root (1);cellular organisms (131567);Bacteria (2)
root (1);cellular organisms (131567);Bacteria (2)
root (1);cellular organisms (131567);Bacteria (2)
root (1);cellular organisms (131567);Bacteria (2)
root (1);cellular organisms (131567);Bacteria (2)
root (1);cellular organisms (131567);Bacteria (2)
root (1);cellular organisms (131567);Bacteria (2)
root (1);cellular organisms (131567);Bacteria (2)

root (1);cellular organisms (131567);Bacteria (2)
root (1);cellular organisms (131567);Bacteria (2);FCB group (1783270);Bacteroidetes/Chlorobi group (68336);Bact
root (1);cellular organisms (131567);Bacteria (2)
root (1);cellular organisms (131567);Bacteria (2)
root (1);cellular organisms (131567);Bacteria (2)
root (1);cellular organisms (131567);Bacteria (2);FCB group (1783270);Bacteroidetes/Chlorobi group (68336);Bact
root (1);cellular organisms (131567);Bacteria (2);FCB group (1783270);Bacteroidetes/Chlorobi group (68336);Bact
root (1);cellular organisms (131567);Bacteria (2);FCB group (1783270);Bacteroidetes/Chlorobi group (68336);Bact
root (1);cellular organisms (131567);Bacteria (2)
root (1);cellular organisms (131567);Bacteria (2);FCB group (1783270);Bacteroidetes/Chlorobi group (68336);Bact
root (1);cellular organisms (131567);Bacteria (2)
root (1);cellular organisms (131567);Bacteria (2)
root (1);cellular organisms (131567);Bacteria (2)
root (1);cellular organisms (131567);Bacteria (2)
root (1);cellular organisms (131567);Bacteria (2);FCB group (1783270);Bacteroidetes/Chlorobi group (68336);Bact
root (1);cellular organisms (131567);Bacteria (2)
root (1);cellular organisms (131567);Bacteria (2)
root (1);cellular organisms (131567);Bacteria (2)
root (1);cellular organisms (131567);Bacteria (2)
root (1);cellular organisms (131567);Bacteria (2)
root (1);cellular organisms (131567);Bacteria (2)
root (1);cellular organisms (131567);Bacteria (2)
root (1);cellular organisms (131567);Bacteria (2);FCB group (1783270);Bacteroidetes/Chlorobi group (68336);Bact
root (1);cellular organisms (131567);Bacteria (2);FCB group (1783270);Bacteroidetes/Chlorobi group (68336);Bact
root (1);cellular organisms (131567);Bacteria (2)
root (1);cellular organisms (131567);Bacteria (2)
root (1);cellular organisms (131567);Bacteria (2)
root (1);cellular organisms (131567);Bacteria (2);FCB group (1783270);Bacteroidetes/Chlorobi group (68336);Bact
root (1);cellular organisms (131567);Bacteria (2);Terrabacteria group (1783272)
root (1);cellular organisms (131567);Bacteria (2)
root (1);cellular organisms (131567);Bacteria (2)
root (1);cellular organisms (131567);Bacteria (2)
root (1);cellular organisms (131567);Bacteria (2)
root (1);cellular organisms (131567);Bacteria (2)
root (1);cellular organisms (131567);Bacteria (2)
root (1);cellular organisms (131567);Bacteria (2)
root (1);cellular organisms (131567);Bacteria (2)
root (1);cellular organisms (131567);Bacteria (2)
root (1);cellular organisms (131567);Bacteria (2)
root (1);cellular organisms (131567);Bacteria (2)
root (1);cellular organisms (131567);Bacteria (2)
root (1);cellular organisms (131567);Bacteria (2)
root (1);cellular organisms (131567);Bacteria (2)
root (1);cellular organisms (131567);Bacteria (2)
root (1);cellular organisms (131567);Bacteria (2)
root (1);cellular organisms (131567);Bacteria (2);FCB group (1783270);Bacteroidetes/Chlorobi group (68336);Bact
root (1);cellular organisms (131567);Bacteria (2);FCB group (1783270);Bacteroidetes/Chlorobi group (68336);Bact

root (1);cellular organisms (131567);Bacteria (2);FCB group (1783270);Bacteroidetes/Chlorobi group (68336);Bact
root (1);cellular organisms (131567);Bacteria (2);FCB group (1783270);Bacteroidetes/Chlorobi group (68336);Bact
root (1);cellular organisms (131567);Bacteria (2)
root (1);cellular organisms (131567);Bacteria (2);FCB group (1783270);Bacteroidetes/Chlorobi group (68336);Bact
root (1);cellular organisms (131567);Bacteria (2);Proteobacteria (1224)
root (1);cellular organisms (131567);Bacteria (2)
root (1);cellular organisms (131567);Bacteria (2);Proteobacteria (1224);Gammaproteobacteria (1236);Pseudomor
root (1);cellular organisms (131567);Bacteria (2);Proteobacteria (1224);Gammaproteobacteria (1236);Pseudomor
root (1);cellular organisms (131567);Bacteria (2)
root (1);cellular organisms (131567);Bacteria (2)
root (1);cellular organisms (131567);Bacteria (2)
root (1);cellular organisms (131567);Bacteria (2)
root (1);cellular organisms (131567);Bacteria (2);Proteobacteria (1224);Gammaproteobacteria (1236)
root (1);cellular organisms (131567);Bacteria (2);Proteobacteria (1224);Gammaproteobacteria (1236)
root (1);cellular organisms (131567);Bacteria (2)
root (1);cellular organisms (131567);Bacteria (2)
root (1);cellular organisms (131567);Bacteria (2)
root (1);cellular organisms (131567);Bacteria (2)
root (1);cellular organisms (131567);Bacteria (2)
root (1);cellular organisms (131567);Bacteria (2)
root (1);cellular organisms (131567);Bacteria (2)
root (1);cellular organisms (131567);Bacteria (2)
root (1);cellular organisms (131567);Bacteria (2)
root (1);cellular organisms (131567);Bacteria (2)

:eroidetes (976)
:eroidetes (976)

:eroidetes (976)

:eroidetes (976);Bacteroidia (200643);Bacteroidales (171549)
:eroidetes (976)
:eroidetes (976)

teroidetes (976)

teroidetes (976)

:eroidetes (976);Bacteroidia (200643);Bacteroidales (171549)

:eroidetes (976)
:eroidetes (976);Bacteroidia (200643)
:eroidetes (976);Bacteroidia (200643)

:eroidetes (976);Bacteroidia (200643);Bacteroidales (171549)

:eroidetes (976);Bacteroidia (200643);Bacteroidales (171549)

:eroidetes (976)
:eroidetes (976);Bacteroidia (200643)

:eroidetes (976)

:eroidetes (976)
:eroidetes (976);Bacteroidia (200643);Bacteroidales (171549)

teroidetes (976);Bacteroidia (200643);Bacteroidales (171549)
teroidetes (976);Bacteroidia (200643);Bacteroidales (171549)

teroidetes (976);Bacteroidia (200643);Bacteroidales (171549)

nadales (72274);Pseudomonadaceae (135621);Pseudomonas (286)
nadales (72274);Pseudomonadaceae (135621);Pseudomonas (286)
