# Supplementary material for: High-Throughput Recovery and Characterization of Metagenome-Derived Glycoside Hydrolase-Containing Clones as a Resource for Biocatalyst Development
Source: mSystems. 2019 Jun 4;4(4):e00082-19. doi: 10.1128/mSystems.00082-19 (PMC6550366; doi:10.1128/mSystems.00082-19)
Supplement: TABLE S4 [file mSystems.00082-19-st004.pdf]

Fosmid.Name	GH5s	Subfamilies
40500_12_L11	1	GH5_1
12200_16_F10	2	GH5_2
FOS62_25_O06	1	GH5_25
FOS62_41_L01	1	GH5_25
NapDC_20_D21	1	GH5_26
TolDC_31_E21	1	GH5_26
TolDC_35_I03	1	GH5_26
FOS62_41_I01	2	GH5_4, GH5_36
FOS62_26_K16	1	GH5_37
FOS62_30_J11	1	GH5_37
FOS62_42_D11	1	GH5_37
FOS62_42_K13	1	GH5_37
FOS62_23_J07	1	GH5_38
FOS62_24_J23	1	GH5_38
FOS62_24_L18	1	GH5_38
FOS62_27_P24	1	GH5_38
FOS62_28_A14	1	GH5_38
FOS62_30_L24	1	GH5_38
FOS62_36_J17	1	GH5_38
FOS62_36_K01	1	GH5_38
FOS62_40_E07	1	GH5_38
FOS62_41_K10	1	GH5_38
FOS62_43_J23	1	GH5_38
FOS62_43_O18	1	GH5_38
FOS62_44_A15	1	GH5_38
FOS62_44_E09	1	GH5_38
NO001_07_A13	2	GH5_39
NO001_01_I19	1	GH5_39, GH5_46
SCR03_04_B15	1	GH5_4
TolDC_20_J14	1	GH5_4
CO182_11_I14	1	GH5_45
CO182_24_J12	1	GH5_45
NapDC_53_D04	1	GH5_45
CO004_05_B17	1	GH5_46
FOS62_25_L08	1	GH5_46
FOS62_28_K23	1	GH5_46
NA004_04_B18	1	GH5_46
NO001_03_P09	1	GH5_46
PWCG7_19_I21	1	GH5_46
PWCG7_19_J20	1	GH5_46
TolDC_59_K14	2	GH5_46
CO002_07_L07	2	GH5_39, GH5_46
CB004_07_C21	3	GH5_39, GH5_41, GH5_46

Fosmid.Name	GH30s	Subfamilies
12200_16_F10	1	GH30_1

FOS62_34_D13	2	GH30_8 , GH30_8
FOS62_37_C18	2	GH30_8 , GH30_8
FOS62_37_N12	2	GH30_8 , GH30_8
FOS62_38_G18	2	GH30_8 , GH30_8
FOS62_41_I01	1	GH30_8
FOS62_43_C07	2	GH30_8 , GH30_8
FOS62_43_J20	2	GH30_8 , GH30_8
FOS62_46_L17	2	GH30_8 , GH30_8
PWCG7_19_I21	1	GH30_3
PWCG7_19_J20	1	GH30_3
