# Supplementary material for: High-Throughput Recovery and Characterization of Metagenome-Derived Glycoside Hydrolase-Containing Clones as a Resource for Biocatalyst Development
Source: mSystems. 2019 Jun 4;4(4):e00082-19. doi: 10.1128/mSystems.00082-19 (PMC6550366; doi:10.1128/mSystems.00082-19)
Supplement: TEXT S1 [file mSystems.00082-19-s0001.docx]

*p*-Nitrophenyl β-D-galactopyranosyl-(1**
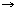
**2)-β-D-glucopyranoside :

^1^H NMR (400 MHz, Deuterium Oxide) δ 8.33 – 8.26 (m, 2H), 7.32 – 7.23 (m, 2H), 5.49 (d, *J* = 7.5 Hz, 1H, H-1), 4.80 (d, *J* = 7.5 Hz, 1H, H-1’), 3.98 – 3.89 (m, 3H, H-6, H-4’, H-2), 3.84 (pt, *J* = 9.1 Hz, 1H, H-3), 3.80 – 3.68 (m, 3H, H-6, H-5, H-3’), 3.66 – 3.61 (m, 1H, H-5’), 3.60 – 3.53 (m, 3H, H-5, H-4, H-6’a), 3.25 (dd, *J* = 11.2, 6.4 Hz, 1H, H-6’b).

Linkage was determined by ^1^H-^13^C HMBC experiment (Heteronuclear Multiple Bond Correlation) showing correlation between H-1’ (4.80 ppm) and C-2 (81.19 ppm), COSY (homonuclear COrrelation SpectroscopY) experiment showing correlation between H-1(5.49 ppm) and H-2 (3.93 ppm), as well as ^1^H-^13^C HSQC experiment (Heteronuclear Single Quantum Correlation) showing correlation between H-2 (3.93 ppm) and C-2 (81.19 ppm).

*p*-Nitrophenyl β-D-galactopyranosyl-(1**
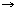
**3)-β-D-glucopyranoside :

^1^H NMR was shown to be identical to that data previously recorded by Faijes et at.[^1^](#_ENREF_1)

*p*-Nitrophenyl β-D-galactopyranosyl-(1**
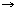
**2)-α-D-xylopyranoside :

^1^H NMR (400 MHz, Deuterium Oxide) δ 8.35 – 8.20 (m, 2H), 7.35 – 7.24 (m, 2H), 6.09 (d, *J* = 3.5 Hz, 1H, H-1), 4.63 (d, *J* = 7.7 Hz, 1H, H-1’), 4.05 (t, *J* = 9.3 Hz, 1H), 3.91 (dd, *J* = 3.6 Hz, 9.1 Hz, 1H, H-2), 3.91 – 3.88 (m, 1H), 3.84 – 3.73 (m, 2H), 3.70 – 3.63 (m, 2H), 3.61 – 3.53 (m, 3H), 3.45 (dd, *J* = 11.4, 7.3 Hz, 1H, H-6’b).

Linkage was determined by ^1^H-^13^C HMBC experiment showing correlation between H-1’ (4.63 ppm) and C-2 (80.3 ppm), COSY experiment showing correlation between H-1(6.09 ppm) and H-2 (3.91 ppm), as well as ^1^H-^13^C HSQC experiment showing correlation between H-2 (3.91 ppm) and C-2 (80.3 ppm).

*p*-Nitrophenyl β-D-galactopyranosyl-(1**
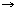
**2)-β-D-xylopyranoside:

^1^H NMR (400 MHz, Deuterium Oxide) δ 8.35 – 8.28 (m, 2H), 7.33 – 7.25 (m, 2H), 5.51 (d, *J* = 6.8 Hz, 1H, H-1), 4.79 (d, *J* = 7.8 Hz, 1H, H-1’), 4.10 (dd, *J* = 11.6, 4.0 Hz, 1H), 3.99 – 3.91 (m, 2H), 3.87 – 3.79 (m, 2H), 3.72 (dd, *J* = 10.0, 3.4 Hz, 1H), 3.67 – 3.56 (m, 3H), 3.29 (dd, *J* = 11.2, 6.4 Hz, 1H H-6’b).

Linkage was determined by ^1^H-^13^C HMBC experiment showing correlation between H-1’ (4.79 ppm) and C-2 (80.72 ppm), COSY experiment showing correlation between H-1(5.51 ppm) and H-2 (3.94 ppm), as well as ^1^H-^13^C HSQC experiment showing correlation between H-2 (3.94 ppm) and C-2 (80.72 ppm).

*p*-Nitrophenyl 6-azido-6-deoxy-β-D-galactopyranosyl-(1**
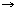
**2)-β-D-glucopyranoside:

^1^H NMR (400 MHz, Deuterium Oxide) δ 8.34 – 8.27 (m, 2H), 7.33 – 7.27 (m, 2H), 5.50 (d, *J* = 7.5 Hz, 1H, H-1), 4.85 (d, *J* = 7.9 Hz, 1H H-1’), 3.98 – 3.90 (m, 2H), 3.88 – 3.87 (m, 1H), 3.84 (t, *J* = 9.2 Hz, 1H), 3.80 – 3.68 (m, 3H), 3.61 – 3.54 (m, 2H), 3.25 – 3.21 (m, 2H, H-6’a and H-6’b).

Linkage was determined by ^1^H-^13^C HMBC experiment showing correlation between H-1’ (4.85 ppm) and C-2 (81.24 ppm), COSY experiment showing correlation between H-1(5.50 ppm) and H-2 (3.92 ppm), as well as ^1^H-^13^C HSQC experiment showing correlation between H-2 (3.92 ppm) and C-2 (81.24 ppm).

*p*-Nitrophenyl 6-azido-6-deoxy-β-D-galactopyranosyl-(1**
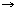
**3)-β-D-glucopyranoside:

^1^H NMR (400 MHz, Deuterium Oxide) δ 8.32 – 8.24 (m, 2H), 7.31 – 7.22 (m, 2H), 5.31 (d, *J* = 7.7 Hz, 1H), 4.77 (s, 1H), 3.99 – 3.83 (m, 5H), 3.83 – 3.77 (m, 1H), 3.77 – 3.60 (m, 5H), 3.51 (dd, *J* = 13.1, 4.0 Hz, 1H, H-6’b).

Linkage was determined by ^1^H-^13^C HMBC experiment showing correlation between H-1’ (4.77 ppm) and C-2 (83.94 ppm), H-2 (3.85 ppm) and C-1 (99.37 ppm), as well as H-2 (3.85 ppm) and C-3 (83.92 ppm).

*p*-Nitrophenyl 6-azido-6-deoxy-β-D-galactopyranosyl-(1**
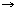
**4)-β-D-glucopyranoside:

^1^H NMR (600 MHz, Deuterium Oxide) δ 8.33 – 8.23 (m, 2H), 7.26 (d, *J* = 9.1 Hz, 2H), 5.31 (d, *J* = 7.8 Hz, 1H, H-1), 4.52 (d, *J* = 7.8 Hz, 1H, H-1’), 4.04 – 3.99 (m, 1H, H-6a), 3.93 (d, *J* = 3.4 Hz, 1H, H-4,), 3.88 – 3.84 (m, 3H), 3.82 – 3.79 (m, 2H), 3.72 – 3.67 (m, 2H), 3.63 (dd, *J* = 13.1, 8.5 Hz, 1H, H-6’a), 3.59 – 3.55 (m, 2H).

Linkage was determined by ^1^H-^13^C HMBC experiment showing correlation between H-1’ (4.52 ppm) and C-4 (78.2 ppm), H-6a (4.00 ppm) and C-4 (78.2 ppm), as well as H-6b (3.86 ppm) and C-4 (78.2 ppm).

Reference:

1. Faijes, M., Saura-Valls, M., Pérez, X., Conti, M. & Planas, A. Acceptor-dependent regioselectivity of glycosynthase reactions by Streptomyces E383A β-glucosidase. *Carbohydrate Research* **341**, 2055-2065 (2006).
